# The CpxR response regulator mediates the virulence of *Klebsiella pneumoniae* by regulating the expression of virulence-associated genes

**DOI:** 10.1128/spectrum.02928-25

**Published:** 2025-11-24

**Authors:** Zhiyuan Liu, Runze Wang, Jiahao Guan, Hong-Yu Ou, Yanjie Chao, Zhaoyan Chen

**Affiliations:** 1 Intensive Care Unit, First Affiliated Hospital of Guangxi Medical University 117742 , Nanning, China; 2 Microbial RNA Systems Biology Unit, Shanghai Institute of Immunity and Infection, Chinese Academy of Sciences 85402 , Shanghai, China; 3 State Key Laboratory of Microbial Metabolism, Joint International Laboratory on Metabolic and Developmental Sciences, School of Life Sciences and Biotechnology, Shanghai Jiao Tong University 553742 , Shanghai, China; South China Sea Institute of Oceanology, Chinese Academy of Sciences, Guangzhou, Guangdong, China

**Keywords:** *Klebsiella pneumoniae*, two-component system, CpxR, virulence factor, transcriptional regulation

## Abstract

*Klebsiella pneumoniae* is a prominent human pathogen that has developed resistance to multiple antibiotics. While the roles of capsules and siderophores are well established, the identification of additional virulence determinants remains limited. In this study, we hypothesize that the two-component system response regulator CpxR is integral to the regulation of *K. pneumoniae* virulence via control of specific virulence-associated genes. The deletion of the *cpxR* gene resulted in reduced serum resistance and attenuated virulence in both *Galleria mellonella* larvae and murine infection models compared to the wild-type strain. To elucidate the repertoire of virulence-associated genes regulated by CpxR, a multidisciplinary workflow was employed, integrating RNA sequencing, real-time quantitative PCR, gene knockout strategies, serum resistance assays, and infection experiments utilizing *Galleria mellonella*. Among the genes identified with significantly diminished expression following *cpxR* deletion, *KPHS_28080* emerged as a novel candidate virulence-associated gene. The deletion of *KPHS_28080* impaired serum survival in both the carbapenem-resistant CRKp HS11286 and the hypervirulent hvKp ATCC43816 strains. Furthermore, the deletion of *KPHS_28080* in hvKp ATCC43816 led to significantly decreased colonization and impaired dissemination to multiple organs in murine models, corresponding with an overall reduction in virulence. The promoter region of *KPHS_28080* harbors a conserved CpxR-binding motif, which enhances promoter activity and gene transcription upon CpxR binding. Sequence alignment revealed that *KPHS_28080* encodes a member of the short-chain dehydrogenase family, and this gene is highly conserved among *K. pneumoniae* strains. These results elucidate the pivotal role of CpxR in mediating virulence in *K. pneumoniae* and clarify its regulatory impact on virulence-associated gene expression.

**IMPORTANCE:**

The mechanisms underlying the pathogenicity of *Klebsiella pneumoniae*, particularly the discovery of novel virulence factor genes, remain poorly understood. CpxR, a response regulator of the two-component system, is critical for mediating envelope stress responses. While CpxR has been implicated in the virulence of diverse bacterial pathogens, its role in *K. pneumoniae* remains elusive. In this study, we demonstrate that CpxR significantly enhances *K. pneumoniae* virulence in both *Galleria mellonella* and murine infection models. Furthermore, we identify a previously uncharacterized virulence-associated gene, encoding a short-chain dehydrogenase/reductase oxidoreductase family member, whose expression is upregulated by CpxR. Given the clinical prevalence and antimicrobial resistance of *K. pneumoniae*, elucidating CpxR-dependent virulence regulation and its novel target provides a pivotal framework for developing therapeutic strategies against these challenging infections.

## INTRODUCTION

*Klebsiella pneumoniae* has emerged as a significant pathogen within the *Enterobacteriaceae* family, characterized by its multidrug resistance and potential for hypervirulence. It is capable of inducing a range of diseases (1), including liver abscesses, pneumonia, and bacteremia (2). The virulence determinants of this organism have primarily included capsules, siderophores, fimbriae, and lipopolysaccharide (LPS) (3). The main distinctions between classic *K. pneumoniae* and hypervirulent *K. pneumoniae* (hvKp) are found in their capsule structures and siderophore profiles (4), with the genes responsible for hypervirulent traits predominantly located on virulence plasmids (5). Recent investigations have identified novel chromosomal virulence factors (6), such as the Type VI Secretion System (7). However, there remains a considerable gap in the comprehensive research of screening and discovering virulence determinants of *K. pneumoniae*.

CpxAR is a bacterial two-component system (TCS) monitoring the inner-membrane homeostasis (8, 9), with CpxA as a cell membrane protein histidine kinase and CpxR as a cytosolic protein response regulator (RR). Upon activation, CpxR interacts with the promoter regions of target genes. Importantly, CpxR has been implicated in the virulence of various pathogenic bacteria (10–14). For instance, the deletion of *cpxR* in *Escherichia coli* results in diminished virulence (15); however, the underlying mechanisms remain poorly elucidated. CpxR is known to enhance the expression of critical virulence genes, such as biofilm-associated genes like *rpoE* and *pgaC* in *K. pneumoniae* (16), as well as *icm* and *dot* in *Legionella pneumophila* (17). Research focusing on the influence of CpxR on the virulence of *K. pneumoniae* is relatively scarce. The limited studies available indicate that CpxR can regulate fimbrial expression in *K. pneumoniae* (18, 19). However, CpxR, as a global regulator, may modulate other unrecognized virulence genes.

Previously, we have reported that CpxR directly interacts with the promoter regions of the *bla*_KPC_ gene and the *tra* operon in an IncFII conjugative plasmid, thereby enhancing their transcriptional activity in the carbapenem-resistant *K. pneumoniae* (20). This interaction contributes to causing increased carbapenem resistance and the spread of the IncF plasmid pKPHS2 and the bla_KPC-2_ resistance gene (21). Consequently, we hypothesize that CpxR may exert a regulatory role over the expression of other chromosomal genes in *K. pneumoniae*, containing virulence-associated genes. In this study, we investigated the impact of CpxR on the virulence of *K. pneumoniae*. By establishing a robust methodology for screening CpxR-regulated virulence-associated genes, we identified a novel and widely disseminated virulence-associated gene *KPHS_28080* in *K. pneumoniae*.

## MATERIALS AND METHODS

### Strains, plasmids, and primers

All strains and plasmids used in this study are listed in Table S1. All primers used in this study are listed in Table S2. All deletion mutants were generated by λ-red recombination and allelic exchange using the suicide vector pKOBEG-Apr as described in Bi et al. (22). PCR was conducted using a specific forward primer and a reverse primer designed within the gene deletion region. Successful mutant construction was verified by PCR amplification of the target region in the wild-type (WT) strain, with the absence of amplification in the mutant. For complementation assays, the low-copy-number vector pXG10 (1–5 copies per cell) carrying the respective genes was used. All *E. coli* and *K. pneumoniae* strains were cultured in lysogeny broth (LB) medium or on LB agar with appropriate antibiotics.

### Bacterial growth curve assay

After overnight culture, bacterial strains were subcultured (1:100 dilution) in fresh LB medium until the optical density at 600 nm (OD_600 nm_) reached 0.5. Cultures were then diluted 100-fold and transferred to a 96-well microplate (200 µL/well). OD_600 nm_ was monitored at 15 min intervals for 12 hours at 37°C using Bioscreen C° Pro Microplate Absorbance Reader, with continuous shaking. Each strain was assayed in triplicate technical replicates.

### Construction of gene knockout strains

Overnight cultures of the target gene knockout strains (*E. coli* or *K. pneumoniae*) harboring the pKOBEG-Apr plasmid were grown at 30°C in LB medium supplemented with 50 µg/mL apramycin. The cultures were diluted 1:100 in fresh LB medium and grown at 30°C until reaching an OD_600 nm_ of 0.2. λ-Red recombinase expression was induced by adding L-(+)-arabinose to a final concentration of 0.2% (wt/vol), followed by induction for 2 hours at 30°C. Bacterial cultures were then harvested and made electrocompetent. DNA fragments used for gene knockout were amplified by splicing overlap extension PCR using gene-specific primers (Table S2). Electroporation of DNA fragments into bacterial strains was performed using a 0.2 cm cuvette (200 Ω, 25 µF, and 2.5 kV). After recovery, transformants were selected on LB agar containing 200 µg/mL hygromycin overnight at 37°C. Hygromycin-resistant colonies were screened by colony PCR to verify replacement of the target gene with the hygromycin resistance gene (*hph*). Electrocompetent cells prepared from a confirmed mutant were subsequently transformed with the pFLP2-Apr plasmid (expressing FLP recombinase) under identical electroporation conditions. Transformants were selected on LB agar with 50 µg/mL apramycin (37°C, overnight). A single colony was streaked onto LB agar containing 6% (wt/vol) sucrose and incubated at 37°C to counter-select against pFLP2-Apr plasmid (via *sacB*-mediated lethality). Final mutants were verified by replica plating and to confirm excision of the *hph* cassette and plasmid curing.

### Serum resistance assay

Tested bacterial strains were cultured to the stationary phase, centrifuged, washed three times with phosphate-buffered saline (PBS), and resuspended in PBS buffer. The bacterial suspension was adjusted to OD_600 nm_ = 0.5 and then diluted 1,000-fold with PBS for subsequent use. For mouse serum preparation, 6- to 8-week-old C57BL/6 mice were anesthetized with tribromoethanol (20 µL/g). After loss of the righting reflex, the mice were placed supine on a bench, and the chest skin was disinfected with 75% ethanol. A 1 mL syringe with a 22-gage needle was used to aspirate 50 µL of 100 U/mL heparin sodium. Fresh blood was centrifuged at 6,000 × *g* for 10 min at 4°C, and the supernatant was immediately used for experiments. A 5 µL aliquot of the bacterial suspension was mixed with 95 µL of serum or 95 µL of PBS, followed by incubation at 37°C for 6 hours. Serum tolerance was evaluated based on the survival rate, calculated as follows:


(1) $$\text{ Survival rate}=\frac{CFU\left(serum\right)}{CFU\left(PBS\right)}\times 100\%.$$

### Evaluation of *K. pneumoniae* virulence using the *Galleria mellonella* larvae model

Bacterial strains were cultured to the stationary phase, harvested by centrifugation, and washed three times with PBS before resuspension in PBS buffer. Healthy, uniformly yellow larvae (300 ± 20 mg) were selected for experiments. Before inoculation, the larval surface, particularly the prolegs, was disinfected with 75% medical alcohol swabs. After air drying, 10 µL of bacterial suspension was injected into the fourth pair of left posterior prolegs using a microliter syringe. Larvae were then re-disinfected with alcohol swabs, placed in sterile Petri dishes lined with filter paper, and incubated at 37°C. Mortality was monitored at 24-hour intervals until 72 hours post-infection.

### Evaluation of *K. pneumoniae* virulence using the mouse model

Test strains were cultured to the stationary phase, harvested by centrifugation, washed three times with PBS, and resuspended in PBS buffer. Bacterial suspensions were adjusted to OD_600 nm_ = 1.0 and serially diluted 10-fold four times. Two strains were mixed prior to dilution. All experimental mice (BALB/c strain, female, 6–8 weeks, 17–19 g) were obtained from Shanghai Shengchang Biotechnology Co., Ltd. (Shanghai, China) and randomized into groups. Mice were weighed and anesthetized with tribromoethanol (20 µL/g body weight). Upon complete loss of consciousness and righting reflex, mice were held vertically, and 25 µL of bacterial suspension was instilled intranasally using a blunt-ended pipette tip. Control mice received sterile PBS. Mice were monitored at least twice daily for signs of severe distress or meeting predefined emergency euthanasia criteria: >20% body weight loss (relative to acclimation baseline), uncontrollable hemorrhage (>0.5 mL/min), or unresponsiveness to pain stimuli (≥3 consecutive negative toe-pinch responses); mice meeting any criterion were immediately euthanized via CO_2_ overdose followed by cervical dislocation confirmation. At 48 hours post-infection, surviving mice underwent terminal euthanasia via CO_2_ overdose (administered at 30% chamber volume displacement/min for 5 min) followed by cervical dislocation confirmation. Organs were then aseptically collected and divided: one half was fixed in 4% paraformaldehyde (PFA) for histology; the remainder was homogenized with steel beads in 500 µL PBS (50 Hz, 4 × 1 min cycles). Homogenates and blood samples were serially diluted and plated for CFU enumeration to determine bacterial loads.

### Strand-specific RNA sequencing

Total bacterial RNA was extracted using the Trizol method, with RNA quality and integrity assessed by agarose gel electrophoresis, NanoDrop spectrophotometer, and Agilent 2100 Bioanalyzer. rRNA was removed from total RNA using the Ribo-Zero rRNA Removal Kit (Illumina). First-strand cDNA was synthesized using random oligonucleotides and SuperScript III, followed by second-strand cDNA synthesis employing DNA Polymerase I and RNase H, where deoxyuridine triphosphate (dUTP) replaced deoxythymidine dtriphosphate (dTTP). Library quality was verified using an Agilent 2100, DNA concentration was measured with PicoGreen, effective concentration was confirmed by real-time quantitative PCR (qPCR), and sequencing was performed on the Illumina NextSeq 500 platform. Bowtie2 (23) was used to build an index based on the *K. pneumoniae* HS11286 genome, followed by the alignment of sequencing reads to the reference genome. Read counts per gene were generated with featureCounts (v2.0.3) (24) by utilizing the genome’s annotated file. Differential expression analysis was performed using DESeq2 (v1.40.2) in R (25). Gene Ontology (GO) Enrichment Analysis was conducted using topGO (http://geneontology.org/) (26). Genes with a Benjamini-Hochberg adjusted *P*-value < 0.05 and absolute log_2_FoldChange > 1 were deemed significant. We specifically identified genes exhibiting log_2_FoldChange < −7 in the Δ*cpxR* mutant strain relative to the WT control.

### Real-time quantitative PCR

Total RNA was isolated using the RNeasy Mini Kit (Qiagen). Then gDNA was removed, and cDNA was produced by PrimeScript RT Reagent Kit with gDNA Eraser (Takara). Finally, qPCR was performed using Hieff UNICON qPCR SYBR Green Master Mix (YEASEN), and *gapA* was used as an internal control. The gene expression level was calculated by the 2^−ΔΔCT^ method.

### Electrophoretic mobility shift assays

Electrophoretic mobility shift assays (EMSAs) were performed with the Chemiluminescent EMSA Kit (Gel Shift; Beyotime, Catalog #GS009) following the manufacturer’s instructions. EMSAs were carried out by mixing wild type or mutated CpxR at a range of concentrations with EMSA/Gel-Shift Binding Buffer (poly(dI-dC), dithiothreitol [DTT], Glycerol, EDTA, NaCl, MgCl_2_, Tris) at room temperature for 10 min. Then, the 2 pmol promoter DNA probes with a 6-FAM modification at the 5′-end were added and incubated for 20 min. The mixtures were then subjected to electrophoresis in a native 6% polyacrylamide gel with EMSA/Gel-Shift Loading Buffer at 100 V for 60 min.

### β-Galactosidase activity assay

The β-galactosidase activity was quantified based on the hydrolysis of O-nitrobenzene-β-D-galactopyranoside (ONPG). Bacterial cultures grown in LB broth to logarithmic phase (OD_600 nm_ of 0.4–1.0) were pelleted and resuspended in an equal volume of Z buffer (0.06 M Na_2_HPO_4_, 0.04 M NaH_2_PO_4_, 0.01 M KCl, 0.001 M MgSO_4_, and 0.05 M β-mercaptoethanol, pH 7.0). For each assay, 1 mL cell suspension (OD_600 nm_ normalized against Z buffer) was permeabilized with 100 µL chloroform and 50 µL 0.1% (wt/vol) sodium dodecyl sulfate (SDS), followed by 5 min equilibration at 28°C. The reaction was initiated by adding 0.2 mL ONPG (4 mg/mL in Z buffer) and terminated with 500 µL 1 M Na_2_CO_3_ when OD_420 nm_ reached 0.6–1.0. Reaction duration (*T*) was recorded to the nearest second. After centrifugation (12,000 × *g*, 5 min), OD_420 nm_ and OD_550 nm_ of the supernatant were measured. Activity units (U) were calculated as follows.


(2) $$\text{Miller units}=1,000\;\times \;\frac{OD_{420\;nm}-OD_{550\;nm}\times \;1.75}{T\;\times \;OD_{600\;nm}}.$$

### Statistical analysis

The data on the promoter activity, growth curve, and qPCR were derived from three independent assays. The unpaired, two-sided Student’s *t*-test was performed using the R package (https://www.r-project.org/). Statistical significance was considered when *P* ≤ 0.05. * Indicates *P* < 0.05; ** indicates *P* < 0.01; *** indicates *P* < 0.001. All experiments and analyses were conducted from November 2022 to June 2025.

## RESULTS

### Deletion of *cpxR* attenuates the virulence of *K. pneumoniae*

**Fig 1:**
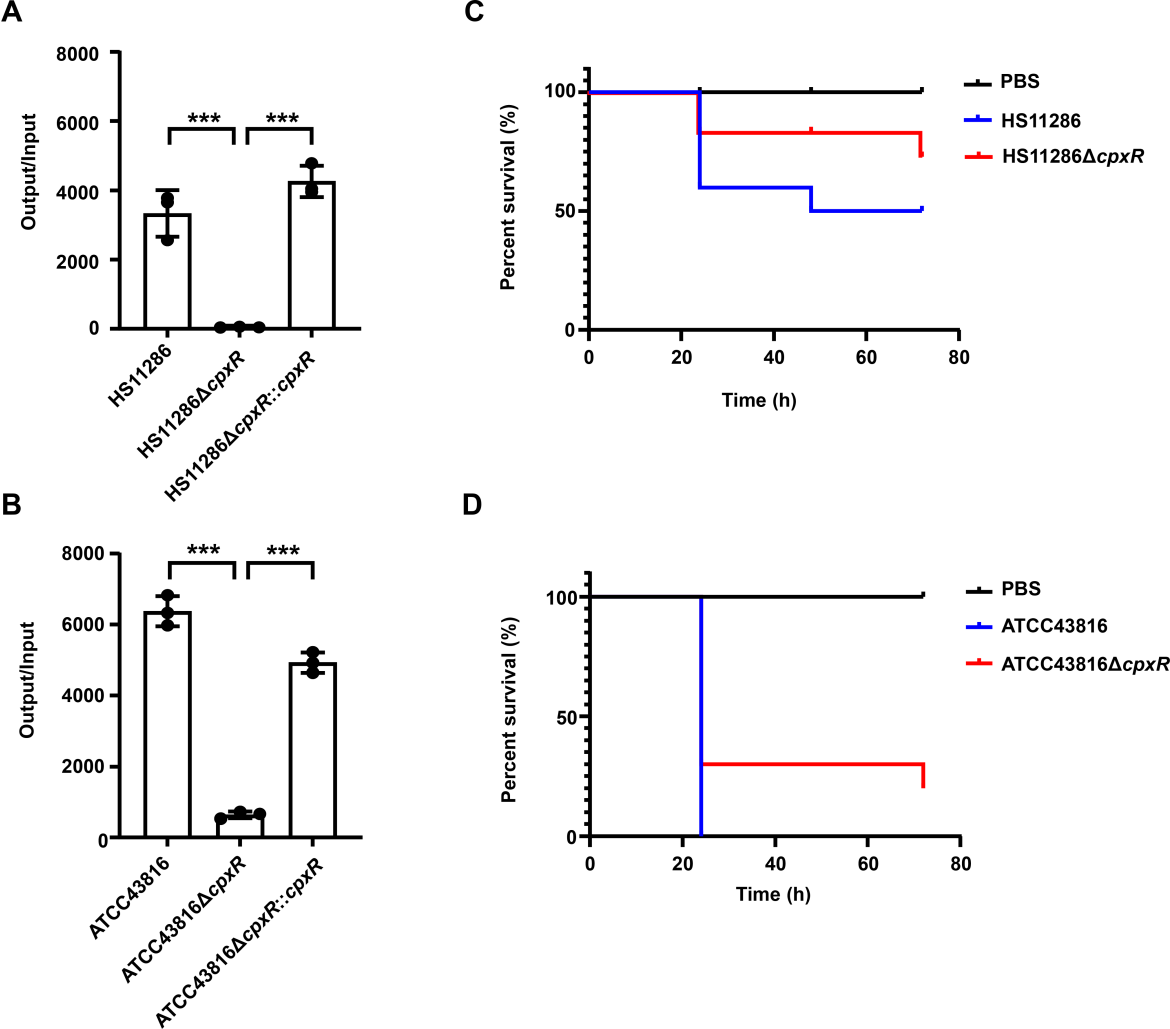
Deletion of *cpxR* attenuates the serum resistance and virulence of *K. pneumoniae* in *Galleria mellonella* larvae. (**A**) Serum resistance of the WT strain CRKp HS11286, the Δ*cpxR* mutant, and the complemented strain Δ*cpxR::cpxR*. After a 6-hour incubation of the strain in serum at 37°C, the ratio of bacterial load to the initial inoculum was quantified. (**B**) Serum resistance of the WT strain hvKp ATCC43816, the Δ*cpxR* mutant, and the Δ*cpxR::cpxR* strain. (**C**) Survival curve for *Galleria mellonella* larvae (*n* = 10) infected with WT strain and the Δ*cpxR* mutant of CRKp HS11286, administered at a dosage of 1.35 × 10^7^ CFU. (**D**) Survival curve for *Galleria mellonella* larvae (*n* = 10) infected with WT strain and the Δ*cpxR* mutant of hvKp ATCC43816, administered at a dosage of 2.4 × 10^5^ CFU. The unpaired, two-sided Student’s *t*-test was performed using the R package (https://www.r-project.org/). Statistical significance was considered when *P* ≤ 0.05. * Indicates *P* < 0.05; ** indicates *P* < 0.01; *** indicates *P* < 0.001.

To elucidate the role of CpxR in the virulence of *K. pneumoniae*, we generated *cpxR* deletion mutants as well as complementary strains of HS11286 (ST11, CRKp), ATCC43816 (ST493, hvKp), and RJ9299 (ST859, hv-CRKp; Table S1). First, we assessed the serum resistance of these *K. pneumoniae* strains. The results for HS11286 (Fig. 1A), ATCC43816 (Fig. 1B), and RJ9299 (Fig. S1) indicated that the deletion of *cpxR* significantly diminished serum resistance compared to the wild-type strains, as demonstrated by a reduced output/input ratio of Δ*cpxR* mutants in serum. Restoration of serum resistance was observed upon complementation of the *cpxR* gene (Δ*cpxR::cpxR*). Next, *Galleria mellonella* larvae were subjected to infection with the wild-type *K. pneumoniae* strains and Δ*cpxR* mutants of HS11286 (Fig. 1C), RJ9299 (Fig. S2), and ATCC43816 (Fig. 1D) at specific dosages. The results demonstrated that all three groups of larvae infected with Δ*cpxR* mutants exhibited higher survival compared to those infected with wild-type strains after 24, 48, and 72 hours.

**Fig 2:**
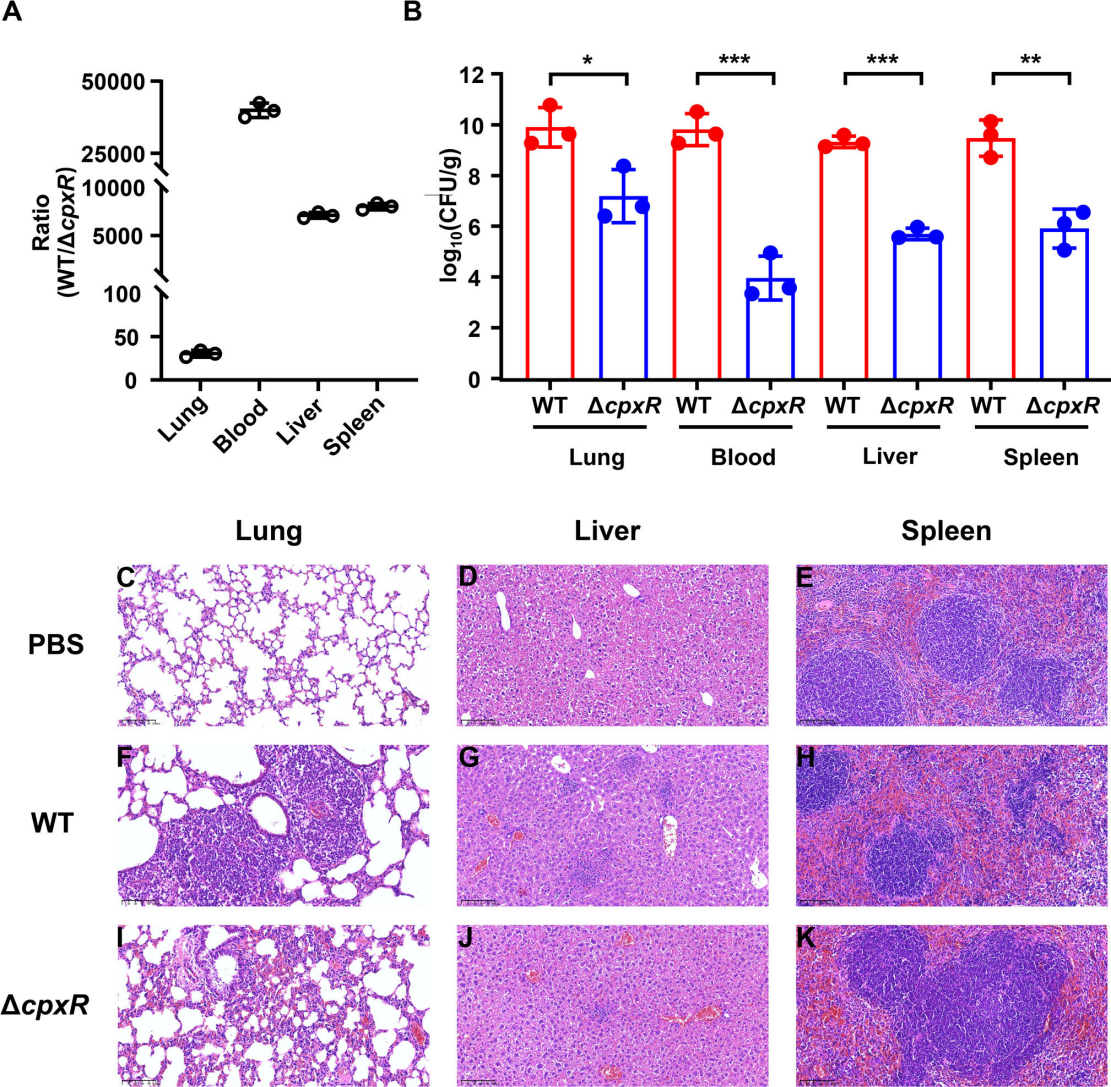
Deletion of *cpxR* attenuates the virulence of *K. pneumoniae* in mice. (**A**) Competition between the WT strain of hvKP ATCC43816 and its Δ*cpxR* mutant in mice. Each mouse was infected with equal amounts of WT and Δ*cpxR* (10^3^ CFU in total). (**B**) Organ colonization of WT and Δ*cpxR* in mice. (**C–K**) Organ pathological changes of mice infected with PBS buffer (**C–E**), WT (**F–H**), and Δ*cpxR* (**I–K**). After intranasal infection with a dose of 10^3^ CFU for 48 hours, euthanasia was performed via cervical dislocation. The liver, lung, and spleen tissues were collected and stained with hematoxylin-eosin (HE). The unpaired, two-sided Student’s *t*-test was performed using the R package (https://www.r-project.org/). Statistical significance was considered when *P* ≤ 0.05. * Indicates *P* < 0.05; ** indicates *P* < 0.01; *** indicates *P* < 0.001.

The virulence of *K. pneumoniae* was further evaluated in a murine model (Fig. 2). Competitive analysis of the wild-type hvKP strain ATCC43816 (ATCC43816WT) to the Δ*cpxR* mutant (ATCC43816Δ*cpxR*) across various organs (lung, blood, liver, and spleen) indicated that ATCC43816Δ*cpxR* was outcompeted by ATCC43816WT in mice (Fig. 2A). Furthermore, the ATCC43816Δ*cpxR* mutant demonstrated significantly reduced colonization capabilities in lung, liver, and spleen tissues compared to the wild-type strain (Fig. 2B). Histopathological examinations revealed distinct tissue responses across the experimental groups. The infection with ATCC43816WT resulted in considerable structural damage to lung tissue, marked by extensive inflammatory exudation, collapsed alveoli, and loss of alveolar integrity (Fig. 2C through K). The liver showed signs of dilated sinusoids, microabscess formation, and necrotic foci, while the spleen presented with atrophy of the white pulp and poorly defined pulp boundaries alongside an accumulation of macrophages. In contrast, the tissues infected with the ATCC43816Δ*cpxR* exhibited comparatively mild pathological changes: lungs retained clearer architecture with thickened septa and localized inflammation; liver hepatocyte arrangement was preserved with minor inflammatory aggregates; and spleen maintained red-white pulp boundaries with marginal zone integrity, despite increased macrophages. These observations underscored the reduced virulence associated with the Δ*cpxR* mutant (Fig. 2C through K). Moreover, it was determined that the deletion of *cpxR* does not influence the growth of HS11286, RJ9299, and ATCC43816 (Fig. S3), thereby eliminating the potential confounding effect of *cpxR* on bacterial growth as a mechanism for virulence modulation. Collectively, these results proved that the CpxR is required for the virulence of *K. pneumoniae.*

### Identification of CpxR-regulated virulence-associated gene *KPHS_28080* via RNA-seq and *in vivo* and *in vitro* models

Given that CpxR acts as a global transcriptional regulator of virulence in *K. pneumoniae*, we developed a workflow for screening virulence-associated genes regulated by CpxR (Fig. S4), including RNA sequencing (RNA-seq), qRT-PCR, serum resistance assays, and the *Galleria mellonella* larvae infection model.

We conducted RNA sequencing to analyze the transcriptomic profiles of the wild-type strain in comparison to the Δ*cpxR* mutant in HS11286. The differentially expressed genes between the Δ*cpxR* and wild-type strains of HS11286 are depicted in a volcano plot (Fig. S5A) and the GO enrichment analysis result (Fig. S6). Among 2,062 differentially expressed genes (Fold-change > 2, *P* < 0.05), seven chromosomal genes (*KPHS_06860*, *KPHS_15890*, *KPHS_16110*, *KPHS_16120*, *KPHS_20300*, *KPHS_28060*, and *KPHS_28080*) were drastically downregulated in the *cpxR* mutant by more than 100-fold (Table S3), indicating that their transcription may be activated by CpxR. The effect of CpxR on these seven genes was confirmed by qRT-PCR (Fig. S5B through H). The Δ*cpxR* mutant demonstrated reduced gene expression compared to the WT strain, whereas the complemented strain (Δ*cpxR::cpxR*) showed restored expression levels. The transcriptional activity of these genes decreased approximately threefold (*KPHS_16110*) to eightfold (*KPHS_28080*), which aligns with the RNA-seq findings. These results reinforce the assertion that CpxR plays a positive role in the transcription of these seven chromosomal genes in *K. pneumoniae*.

**Fig 3:**
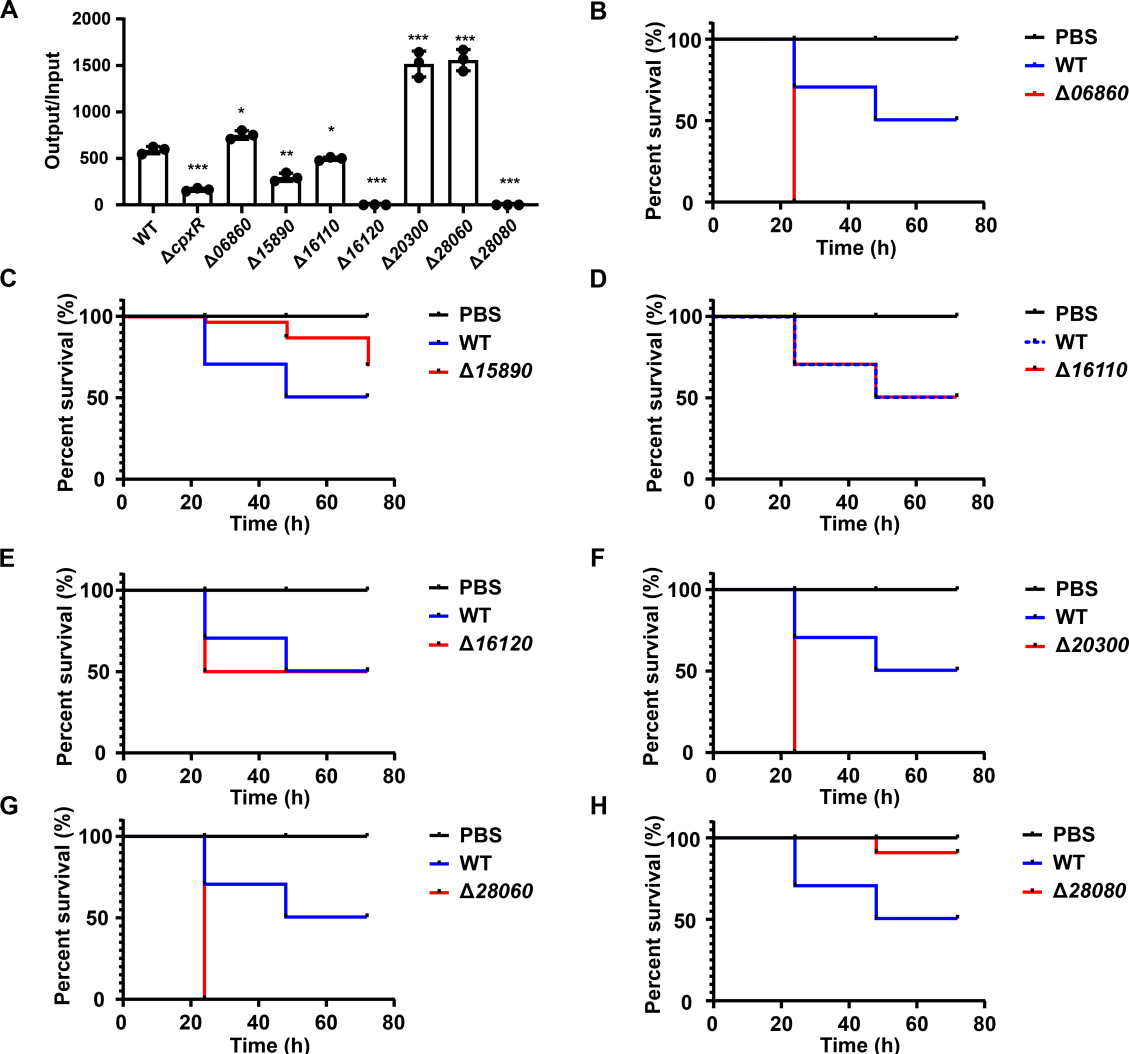
Examination of the influence of CpxR-upregulated genes on the virulence of *K. pneumoniae*. (**A**) Serum resistance of the WT strain of CRKp HS11286 and the seven deletion mutants corresponding to candidate virulence genes. After incubating in serum, the ratio of bacterial load to the initial inoculum was quantified. (**B–H**) Survival curves for *Galleria mellonella* larvae (*n* = 10) infected with HS11286 WT and the deletion mutants, administered at a dosage of 1.35 × 10^7^ CFU. The unpaired, two-sided Student’s *t*-test was performed using the R package (https://www.r-project.org/). Statistical significance was considered when *P* ≤ 0.05. * Indicates *P* < 0.05; ** indicates *P* < 0.01; *** indicates *P* < 0.001.

To understand the contribution of these seven genes in virulence, we removed these genes individually and generated seven deletion mutants, designated as Δ*06860*, Δ*15890*, Δ*16110*, Δ*16120*, Δ*20300*, Δ*28060*, and Δ*28080*. We evaluated the serum resistance of these mutants in comparison to the wild-type strain HS11286 (Fig. 3A). Notably, the Δ*06860*, Δ*20300*, and Δ*28060* mutant strains exhibited a marked enhancement in serum resistance. Conversely, the Δ*16120* and Δ*28080* were incapable of surviving in serum, with bacterial counts declining below the detection limit after 6 hours of incubation in serum.

We next employed the *Galleria mellonella* larvae infection model to evaluate the virulence of various mutants (Fig. 3B through H). At an inoculation dose of 1.35 × 10⁷ CFU, the wild-type strain HS11286 exhibited a survival rate of 50% after 72 hours (LD_50_). In contrast, the Δ*15890* mutant showed an improved survival rate of 70%. The deletion of the *KPHS_16110* or *KPHS_16120* genes resulted in survival rates of 50% for larvae, which were comparable to those of the wild-type strain. Notably, the larvae infected with the mutants Δ*06860*, Δ*20300*, or Δ*28060* mutants exhibited complete mortality within 24 hours, which indicated that these genes may function as potential virulence-repressing factors that are upregulated by CpxR. Conversely, the deletion of the *KPHS_28080* gene significantly diminished the virulence of HS11286 to *Galleria mellonella* larvae, resulting in a survival rate of up to 90% after 72 hours of infection. These results are consistent with serum resistance assays, indicating that KPHS_28080 has a positive effect on the virulence of *K. pneumoniae*.

### Deletion of *KPHS_28080* reduces the virulence of *K. pneumoniae* in mice

**Fig 4:**
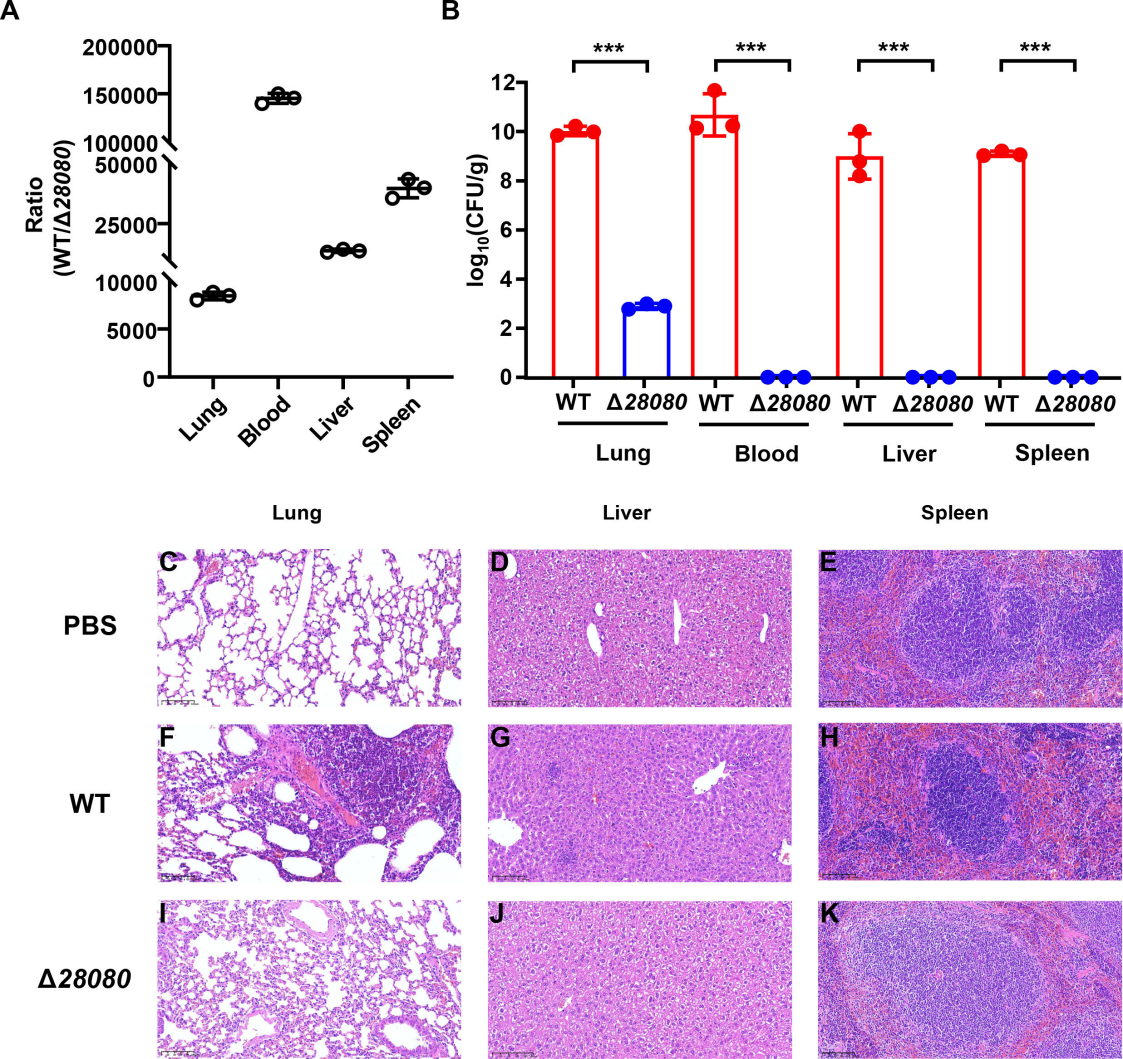
Deletion of *KPHS_28080* reduces the virulence of *K. pneumoniae*. (**A**) Competition between the WT strain of hvKP ATCC43816 and the Δ*28080* mutant in mice. Each mouse was infected with WT and Δ*cpxR* (10^3^ CFU in total). (**B**) Organ colonization of ATCC43816 WT and Δ*cpxR* in mice. (**C–K**) Organ pathological changes of mice infected with PBS buffer (**C–E**), WT (**F–H**), and Δ*28080* (**I–K**). After intranasal infection with a dose of 10^3^ CFU for 48 hours, euthanasia was performed via cervical dislocation. The liver, lung, and spleen tissues were collected and stained with hematoxylin-eosin (HE). The unpaired, two-sided Student’s *t*-test was performed using the R package (https://www.r-project.org/). Statistical significance was considered when *P* ≤ 0.05. * indicates *P* < 0.05; ** indicates *P* < 0.01; *** indicates *P* < 0.001.

To further elucidate the role of *KPHS_28080* in *K. pneumoniae* virulence in mammals, the 1:1 mixture of ATCC43816WT and ATCC43816 *KPHS_28080* deletion mutant (ATCC43816Δ*28080*) was administered intranasally to mice. The results demonstrated the substantial colonization by *K. pneumoniae* across various organs (Fig. 4). Notably, ATCC43816WT predominated over ATCC43816Δ*28080*, with organ-specific ratios recorded as follows: lungs (8,452:1), blood (145,151:1), liver (13,642:1), and spleen (39,572:1; Fig. 4A). Subsequently, mice were intranasally infected with either ATCC43816WT or ATCC43816Δ*28080* at equal doses (1 × 10³ CFU). After 48 hours, bacterial counts in lung, blood, liver, and spleen showed that the ATCC43816WT achieved extensive colonization and proliferation across all examined organs (Fig. 4B). In contrast, ATCC43816Δ*28080* was found below detection thresholds in the blood, liver, and spleen, with only marginal colonization observed in the lungs, approximately 10^5^-fold less than the wild-type strain in the same organ.

Histopathological assessments of tissues from mice infected with either ATCC43816WT or ATCC43816Δ*28080* were conducted (Fig. 4C through K). Consistent with the results presented in Fig. 2, the infection with ATCC43816WT resulted in marked inflammatory infiltration in the liver, lung, and spleen. Conversely, lung tissue from mice infected with ATCC43816Δ*28080* displayed minor structural alterations, including thickened alveolar septa, partial alveolar collapse, and localized inflammatory cell infiltration (Fig. 4I). The liver remained largely healthy, with hepatocytes around central veins maintaining orderly arrangement, distinct nuclei, and vacuolated cytoplasm, without significant inflammation (Fig. 4J). While the spleen exhibits expansion of irregular white pulp, obscured red-white pulp demarcation, and reduced marginal zone cells, its cellular changes are significantly less severe than those in spleens infected with ATCC43816WT (Fig. 4K). Moreover, the deletion of *KPHS_28080* did not influence the growth of HS11286 and ATCC43816 (Fig. S7), which excludes the possibility that KPHS_28080 modulates virulence by controlling bacterial growth. These findings indicate that *KPHS_28080* critically contributes to *K. pneumoniae* virulence in mice, with its deletion leading to a profound reduction in virulence.

### CpxR binds to the promoter region of *KPHS_28080* and enhances its transcription

**Fig 5:**
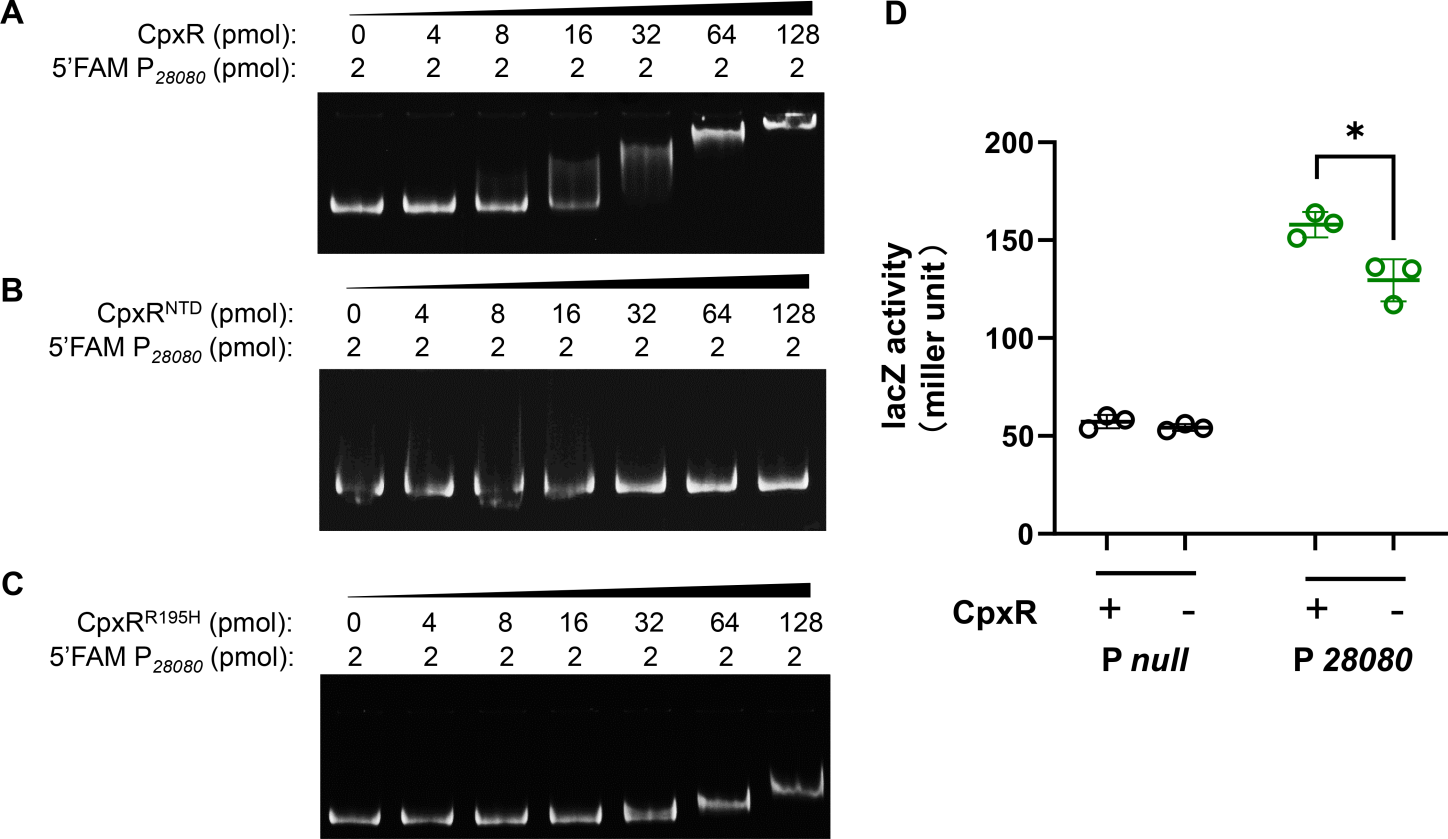
CpxR binds to the promoter region of *KPHS_28080* and enhances its transcription. (**A–C**) EMSAs of increasing amounts of WT CpxR, CpxR^NTD^ (CpxR missing N-terminal domain), and CpxR^R195H^ with 2 pmol of FAM-labeled promoter of *KPHS_28080*. The result of the negative control is available in Fig. S8. (**D**) The activity of *lacZ* reporter fused to the respective promoter was determined in the presence or absence of *cpxR.* The null promoter was used as a negative control. This experiment was performed in *E. coli* DH5ɑΔ*cpxR*, in which the *K. pneumoniae cpxR* gene was provided on a low-copy plasmid. The unpaired, two-sided Student’s *t*-test was performed using the R package (https://www.r-project.org/). Statistical significance was considered when *P* ≤ 0.05. * Indicates *P* < 0.05.

The conserved CpxR binding motif, GTAAA-N_5_-GCAAA (27), was identified within the promoter region of *KPHS_28080*. To elucidate the precise mechanisms through which CpxR enhances the transcription of *KPHS_28080*, we performed EMSAs using FAM-labeled promoter DNA probes. These experiments confirmed the direct binding of wild-type CpxR protein to the *KPHS_28080* promoter (Fig. 5A). Competitive EMSA with excess unlabeled probe successfully inhibited this binding (Fig. S8), affirming its specificity. The deletion of the DNA-binding domain (CpxR^NTD^) completely disrupted interaction (Fig. 5B). Additionally, the mutation in the conserved DNA-binding residue Arg195 (CpxR^R195H^) significantly diminished binding affinity, with only marginal shifts observed only at high protein concentrations (64–128 pmol; Fig. 5C).

In addition, the β-galactosidase assay revealed that the binding of CpxR enhanced the activity of the *KPHS_28080* promoter (Fig. 5D), thereby facilitating downstream transcriptional processes. Furthermore, the qPCR analysis of *KPHS_28080* expression in HS11286 strains showed significantly reduced transcript levels (about 90%) in the Δ*cpxR* mutants, with restoration observed following *cpxR* complementation (Fig. S5H). Collectively, these results suggested that CpxR exerted a positive regulatory effect on *KPHS_28080* expression via direct interaction with the promoter.

### *KPHS_28080* is widely distributed and conserved in *K. pneumoniae*

**Fig 6:**
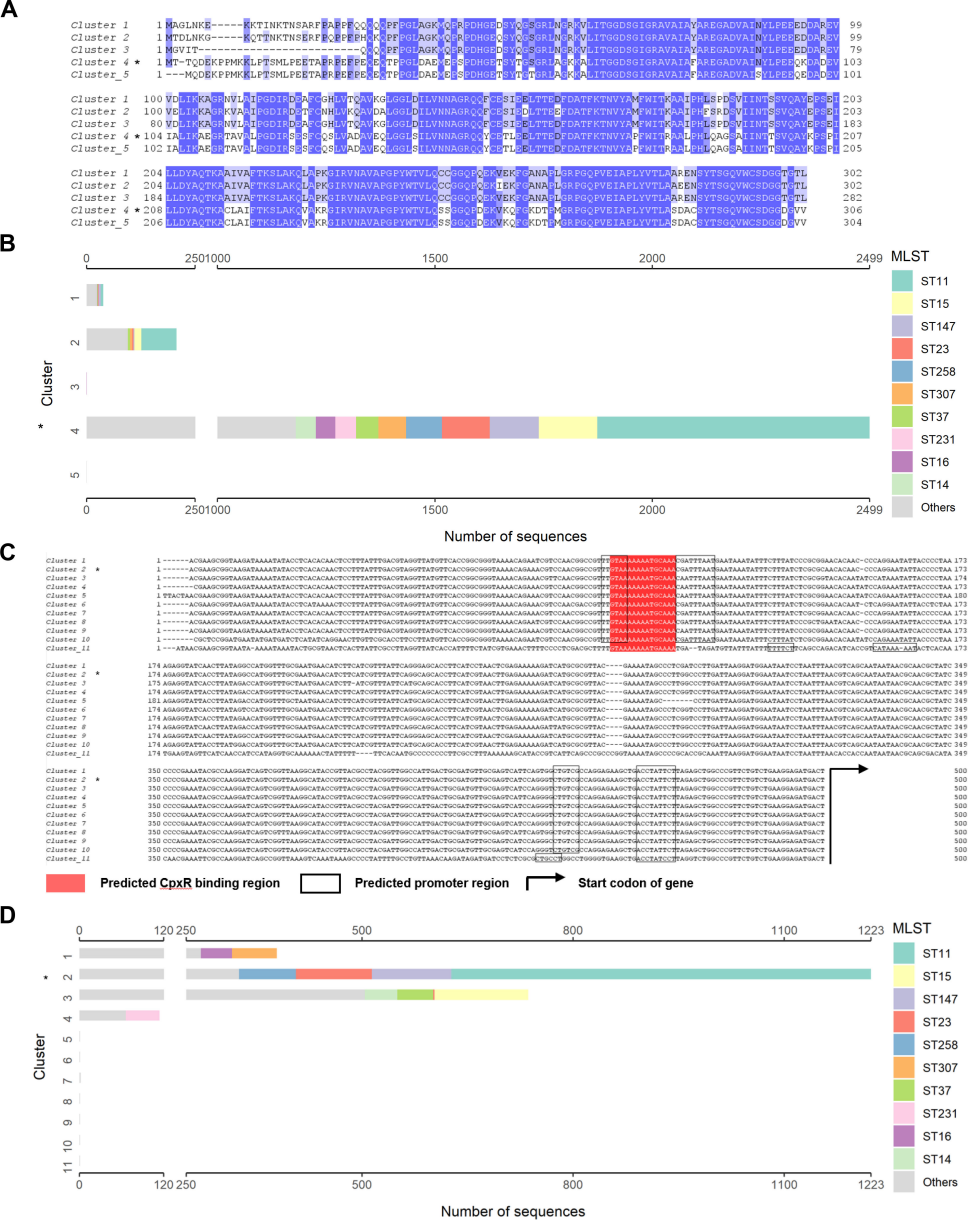
KPHS_28080 is widely distributed and conserved in *K. pneumoniae*. (**A**) Multiple sequence alignments of the KPHS_28080 homologous proteins. (**B**) Distribution of ST types in the clusters of KPHS_28080 homologous proteins. (**C**) Multiple sequence alignments of the clustered DNA sequences 500 bp upstream of the start codons of the *KPHS_28080* homologous genes. (**D**) Distribution of ST types in the clusters of upstream sequences. An asterisk (*) denotes the presence of *KPHS_28080* gene (**B**) or KPHS_28080 protein (**D**) of *K. pneumoniae* HS11286.

Following the identification of *KPHS_28080* as a novel virulence-associated gene regulated directly by CpxR, a comprehensive analysis of KPHS_28080 was conducted in *K. pneumoniae* as well as in other bacteria (Fig. S9). Among 2,539 completely sequenced *K. pneumoniae* strains available in the NCBI RefSeq database by 2 June 2025, 97% (2,463/2,539) of these strains possessed homologous proteins of KPHS_28080. Sequence alignments of these homologous proteins led to the identification of five distinct clusters. Specifically, cluster 4 of KPHS_28080 homologs (*n* = 2,499), which contains KPHS_28080, is predominantly associated with ST11 strains (*n* = 626), followed by ST15 (*n* = 134), ST147 (*n* = 113), and ST23 (*n* = 110; Fig. 6C).

Further analysis of the upstream DNA sequences of *KPHS_28080* homologous genes (available in the NCBI RefSeq database by 2 June 2025) revealed that 90% (2,460/2,747) of these genes contained the classical CpxR-binding motif (GTAAA-N_5_-GCAAA) within their promoter regions. Importantly, variability was observed in the promoter sequences of *KPHS_28080* homologous genes among different sequence types of *K. pneumoniae*. For example, sequences from ST11 (*n* = 596), ST147 (*n* = 113), and ST23 (*n* = 108) align with cluster 2 of *KPHS_28080* homologous genes, whereas sequences from ST15 (*n* = 133) and ST14 (*n* = 46) were classified within cluster 3 (Fig. 6D). Despite this variation, both the CpxR-binding motif and the promoter sequences of *KPHS_28080* homologous genes displayed high conservation across the majority of *K. pneumoniae* strains. Overall, these results demonstrated the widespread distribution and notable conservation of KPHS_28080 in *K. pneumoniae*.

## DISCUSSION

The well-characterized virulence factors of *K. pneumoniae* include the polysaccharide capsule, siderophores, fimbriae, and LPS (28). However, research on novel virulence determinants remains limited. In this study, we demonstrated that the classical TCS RR CpxR plays a significant role in the virulence of *K. pneumoniae*. Furthermore, through screening CpxR-upregulated genes, we identified a novel virulence-associated factor, *KPHS_28080*, which is widely conserved in *K. pneumoniae*.

Utilizing serum resistance assays, *Galleria mellonella* larvae infection models, and murine infection models, we have established the critical role of CpxR in the virulence of *K. pneumoniae*. Initially identified as a key regulator of envelope stress response, CpxR exerts significant influence on the assembly of bacterial surface structures (29), including fimbriae. However, the role of CpxR in influencing the virulence of *K. pneumoniae* has not been definitively established. To our knowledge, this investigation represents the first systematic effort to elucidate the contribution of CpxR to the virulence of *K. pneumoniae* using animal model evidence. Furthermore, we have developed a workflow for screening CpxR-regulated virulence genes utilizing RNA-seq, qPCR, gene deletion, serum resistance assays, and the *Galleria mellonella* larvae infection model. Given that CpxR acts as a global transcriptional regulator, it is plausible that other transcriptional regulators may also be influenced by CpxR’s regulatory network. In *Vibrio cholerae*, ToxR is an important transcriptional regulator associated with virulence, capable of upregulating cholera toxin and toxin-coregulated pilus expression (30). Conversely, the transcriptional activity of *toxR* is also subject to regulation by CpxR (31). Our approach, combining strain-specific RNA-seq for genome-wide transcriptional profiling with *in vivo* and *in vitro* validation, offers a robust strategy for deciphering CpxR’s regulatory mechanisms.

KPHS_28080 protein was annotated as a putative short-chain dehydrogenase/reductase (SDR) (32), which has not traditionally been classified as a virulence factor of *K. pneumoniae*. This study demonstrated its critical role in the virulence of *K. pneumoniae*, influencing bacterial colonization, proliferation, and dissemination across various host organs, mirroring the functional characteristics of our recently identified virulence factor MlaA (33). These findings suggested that novel chromosomally encoded virulence factors contribute to the virulence of *K. pneumoniae*, in addition to capsular polysaccharides. We conducted a manual curation of 1,153 proteins within the SDR family, of which 382 are encoded by bacteria. Phylogenetic analysis showed that the SDR family exhibits extensive distribution not only in *K. pneumoniae* but also across both gram-positive and gram-negative bacteria (Fig. S10). The protein YghA, recognized as an oxidoreductase, plays a role in enabling *Salmonella enterica* to resist reactive oxygen species (ROS), increasing its survivability in host macrophages (34). KPHS_28080 shows a sequence coverage of 91% and identity of 58.84% when aligned with *Salmonella*’s YghA, indicating that KPHS_28080 might possess a similar function in *K. pneumoniae* infection. Further analysis through multiple sequence alignment of proteins within the evolutionary branch containing KPHS_28080 demonstrated KPHS_28080 conservation (Fig. S11). Collectively, KPHS_28080 demonstrates widespread distribution and conservation, potentially contributing to the virulence of *K. pneumoniae* by resisting ROS. Previous research has also indicated that SDR family oxidoreductases in *Mycobacterium tuberculosis* were associated with intermediate metabolism, drug resistance, virulence, and cellular homeostasis (35). When SDR oxidoreductase-deficient strains infect THP-1 cells, they enhance host secretion of pro-inflammatory cytokines, including IL-6, TNF-α, and IL-1β. Although the database for SDR family proteins has been established (36), determining the function of KPHS_28080 remains highly challenging. The broad substrate range within the protein family suggests that even slight variations in sequence may lead to significant differences in substrate specificity (37). The functional characterization of this protein in *K. pneumoniae* remains poorly documented, and the specific mechanisms underlying its virulence-enhancing effects require further investigation.

### Summary

Our investigation revealed that the TCS response regulator CpxR significantly contributes to the virulence of *K. pneumoniae*. Through RNA sequencing, serum resistance assays, and *Galleria mellonella* larvae infection model, we identified a novel virulence-associated gene, *KPHS_28080*, among the genes that are upregulated by CpxR. KPHS_28080 plays a critical role in the virulence of *K. pneumoniae*, facilitating infection and multi-organ dissemination in the murine model, and it exhibits a broad distribution and high conservation across various *K. pneumoniae* strains.

## Data Availability

RNA-seq raw data are available on Gene Expression Omnibus (GEO, GSE300466).

## SUPPLEMENTAL MATERIAL

Supplemental material spectrum.02928-25-s0001.pdf Supplemental methods; Fig. S1 to S11; Table S1 to S3.

ASM does not own the copyrights to Supplemental Material that may be linked to, or accessed through, an article. The authors have granted ASM a non-exclusive, world-wide license to publish the Supplemental Material files. Please contact the corresponding author directly for reuse.
