# Supplementary material for: The CpxR response regulator mediates the virulence of Klebsiella pneumoniae by regulating the expression of virulence-associated genes
Source: Microbiol Spectr. 2025 Nov 24;14(1):e02928-25. doi: 10.1128/spectrum.02928-25 (PMC12772254; doi:10.1128/spectrum.02928-25)
Supplement: Supplemental material — Supplemental methods; Fig. S1 to S11; Table S1 to S3. [file spectrum.02928-25-s0001.pdf]

The CpxR response regulator mediates the virulence of *Klebsiella pneumoniae* by regulating the expression of virulence-associated genes

Supplementary Methods

Construction of pET28a(+)-based wild-type and mutants protein expression plasmids

Expression and purification of proteins

Construction of pXG10-based complementation plasmids

Supplementary Data

Figure S1. Deletion of *cpxR* attenuates the serum resistance of hv-CRKp RJ9299.

Figure S2. Deletion of *cpxR* attenuates the pathogenicity of hv-CRKp RJ9299 in *Galleria mellonella* larvae.

Figure S3. Growth curve of wild-type and Δ*cpxR* mutants of HS11286, RJ9299, and ATCC43816.

Figure S4. Workflow for CpxR-regulated virulence genes screening.

Figure S5. Screening for CpxR up-regulated genes.

Figure S6. Gene Ontology (GO) enrichment analysis between HS11286 wild-type and Δ*cpxR* mutant.

Figure S7. Growth curve of wild-type and Δ*28080* mutants of HS11286 and ATCC43816.

Figure S8. Electrophoretic mobility shift assays negative control.

Figure S9**.** Pipeline for identifying the KPHS_28080 homolog proteins and the CpxR binding sites in the promoter region of *KPHS_28080* homologous genes in the completely sequenced *K. pneumoniae* strains.

Figure S10. Phylogenetic analysis of the bacterial short-chain dehydrogenase/reductase (SDR) family.

Figure S11. Multiple sequence alignment of proteins within the evolutionary branch containing the KPHS_28080 protein.

Table S1. Strains and plasmids used in this study

Table S2. Primers used in this study

Table S3. Seven selected genes with significantly down-regulated expression after CpxR deletion in *K. pneumoniae* HS11286

Supplementary Methods

Construction of pET28a(+)-based mutants protein expression plasmids

For both $CpxR^{NTD}$ and $CpxR^{R195H}$ constructs, the pET28a(+) vector was linearized by digestion with NdeI and HindIII. To generate the $CpxR^{NTD}$ insert (encoding CpxR amino acids 1-131), a DNA fragment was amplified from *K. pneumoniae* HS11286 genomic DNA (gDNA) using primers cpxR-Re-FU-F/R (Table S2). For the $CpxR^{R195H}$ point mutant (Arg195→His), two overlapping DNA fragments containing the mutation site were amplified from *K. pneumoniae* HS11286 gDNA using primer pairs cpxR-pET28-FU-F/cpxR-R195H-R and cpxR-pET28-FU-R/cpxR-R195H-F (Table S2); these fragments were fused via splicing overlap extension PCR (SOE-PCR) to generate the full-length mutant insert. Both the NTD fragment and the R195H SOE-PCR product possessed 15-bp terminal sequences homologous to the ends of the linearized pET28a(+) vector. Each insert was individually ligated into the digested pET28a(+) backbone using Gibson Assembly® (NEB) for seamless cloning, resulting in plasmids pET28a-*cpxR*^{NTD} and pET28a-*cpxR*^{R195H}, respectively.

Expression and purification of proteins

The wild-type *cpxR* gene and its mutation variant genes were cloned into vector pET28a(+) using primers listed in Table S2, and transformed into *E. coli* BL21(DE3). Cells containing plasmids were grown at 37℃. When $OD_{600\ nm}$= 0.5, protein expression was induced overnight at 16°C with 0.2 mM Isopropyl β-D-1-thiogalactopyranoside (IPTG). Subsequently, proteins were purified from the cell lysis supernatant with cobalt metal affinity resin (Takara). Contaminating nucleic acid was removed by HiTrap Heparin HP (GE Healthcare) or HiTrap Capto Q (GE Healthcare).

Construction of pXG10-based complementation plasmids

The linearized pXG10 vector was amplified using primers pXG10-Apr-FU-F/R (Table S2), and the resulting PCR product was digested with DpnI to remove template plasmid DNA. Four distinct DNA fragments were subsequently amplified from *K.*

pneumoniae HS11286 genomic DNA (gDNA) using specific primer pairs (Table S2), overlapping fragments were amplified using primer pairs cpxR-101-FU-F/cpxR-R195H-R and cpxR-101-FU-R/cpxR-R195H-F, which were then fused by splicing overlap extension PCR (SOE-PCR) to yield a product containing the *cpxR* promoter and mutant *cpxR*^{R195H} gene. All amplified fragments possessed 15-bp terminal sequences homologous to the ends of the linearized pXG10 vector. These fragments were individually ligated to the DpnI-digested pXG10 backbone using Gibson Assembly® (NEB), constructing plasmids pXG10-Apr-*cpxR*, pXG10-Apr-*cpxR*^{NTD}, and pXG10-Apr-*cpxR*^{R195H}.

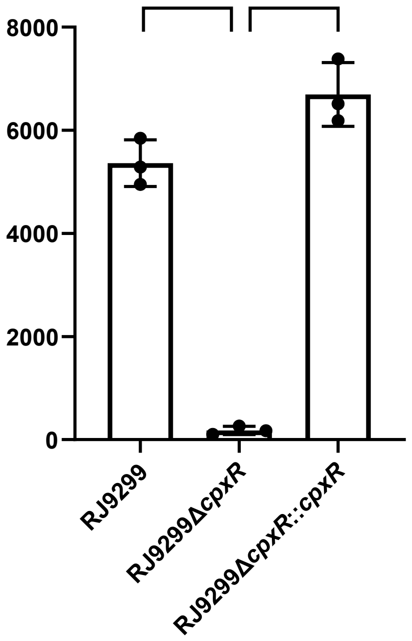

Figure S1. Deletion of *cpxR* attenuates the serum resistance of hv-CRKp RJ9299. After incubating wild-type K. pneumoniae and Δ*cpxR* mutants and complementary strains in serum at 37°C for 6 hours, the ratio of bacterial load to the initial inoculum was determined, respectively. The unpaired, two-sided Student's t-test was performed using the R package (https://www.r-project.org/). Statistical significance was considered when $p \leq 0.05$. * indicates $p < 0.05$; ** indicates $p < 0.01$; *** indicates $p < 0.001$.

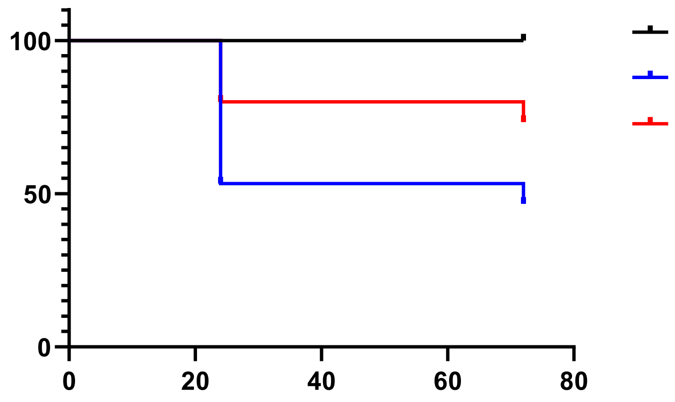

Figure S2. Deletion of *cpxR* attenuates the pathogenicity of hv-CRKp RJ9299 in *Galleria mellonella* larvae. Survival curves for *Galleria mellonella* larvae (*n*=10) infected with wild-type *K. pneumoniae* and Δ*cpxR* mutants of hv-CRKp RJ9299. *Galleria mellonella* larvae were infected at dosage of 1.27×10^4 CFU.

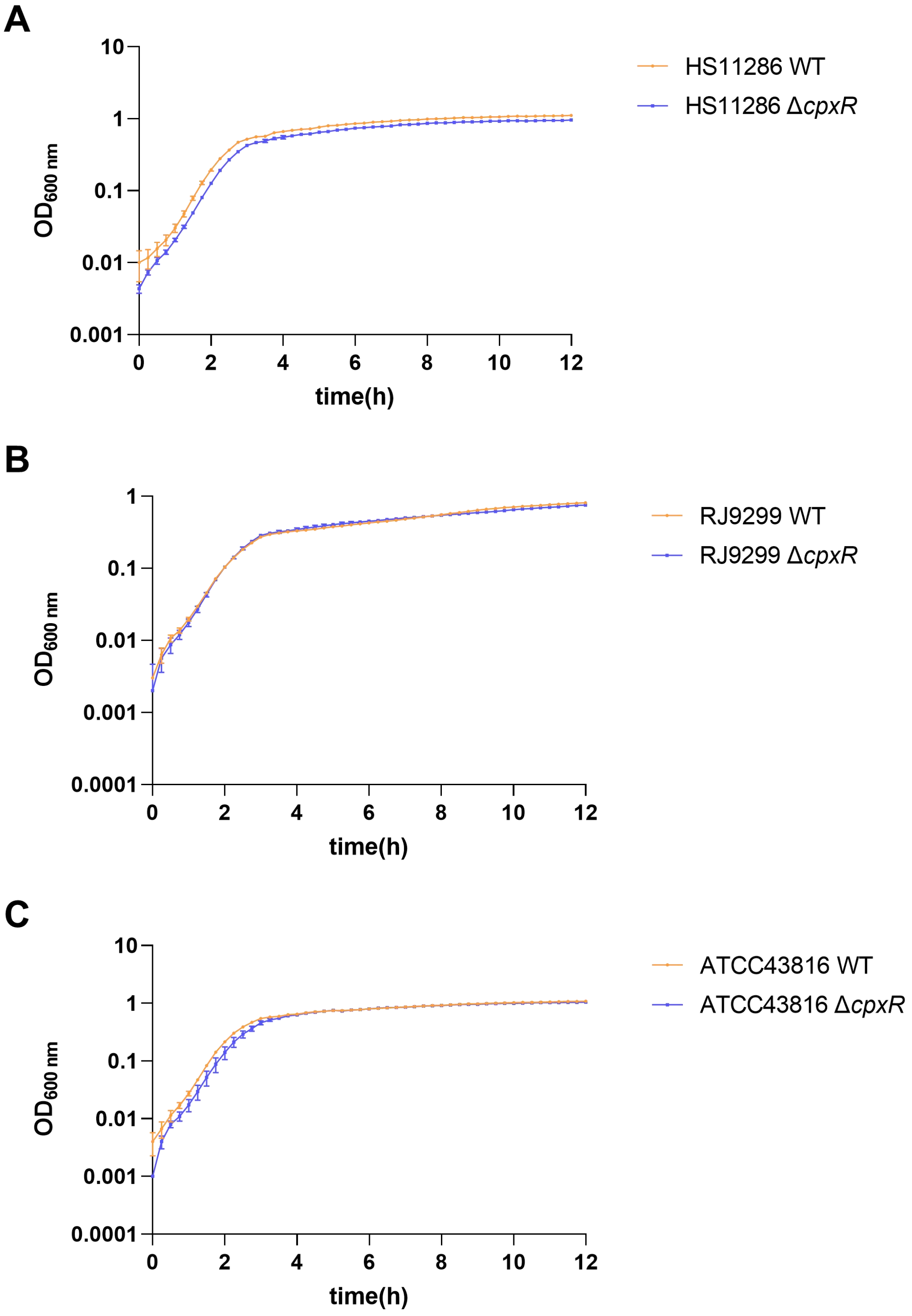

Figure S3. Growth curve of the wild-type and Δ*cpxR* mutant of *K. pneumoniae* HS11286, RJ9299, and ATCC43816.

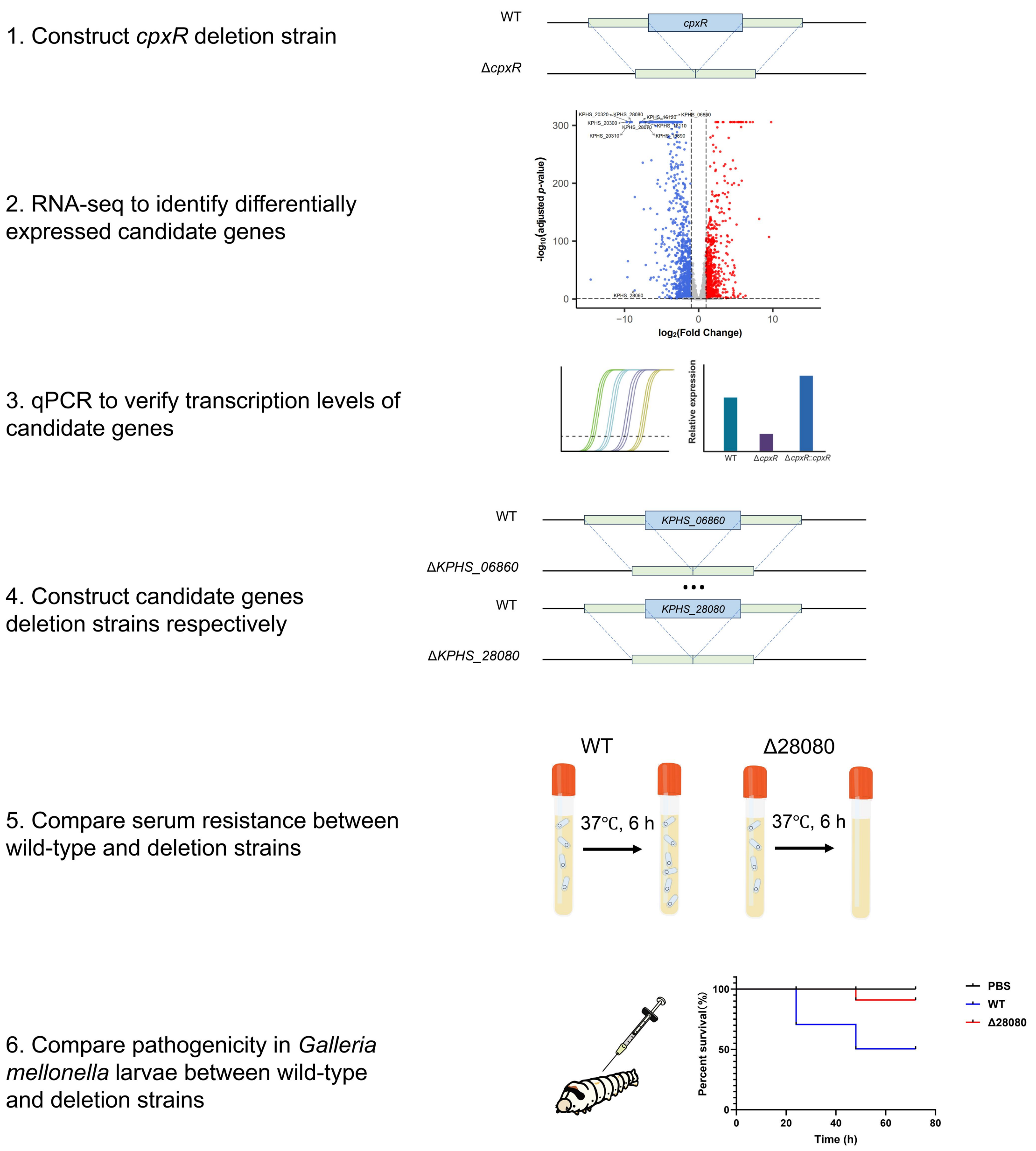

Figure S4. Workflow for screening CpxR-regulated virulence genes. Initially, a *cpxR* deletion strain was constructed. RNA-seq was then performed to identify differentially expressed candidate genes between the wild-type (WT) and Δ*cpxR* strains. Quantitative PCR (qPCR) was used to verify the transcription levels of these candidate genes across WT, Δ*cpxR*, and a complemented strain (Δ*cpxR*::*cpxR*). Subsequently, deletion strains for the candidate genes were constructed. The serum resistance of these deletion strains was compared with that of the WT. Finally, the pathogenicity of the WT and the candidate genes deletion strains was assessed using *Galleria mellonella* larvae. The gene *KPHS_28080* was used as an example for illustration.

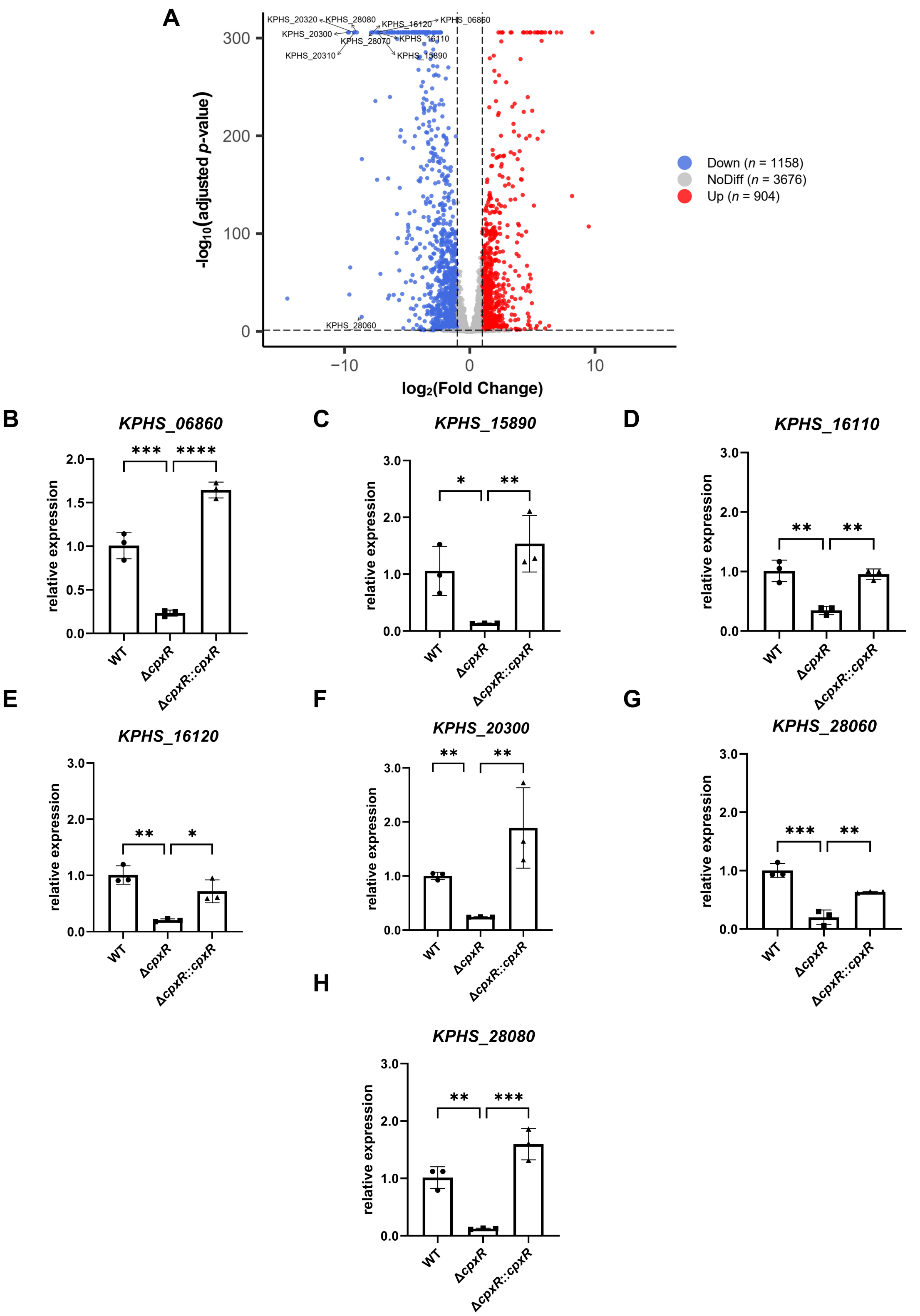

Figure S5. Screening for CpxR up-regulated genes by using RNA-seq and qPCR. (A) Volcano plot of differentially expressed genes comparing Δ*cpxR* to wild-type in HS11286. The x-axis represents $\log_2$FoldChange, and the y-axis represents

$-log_{10}$(adjusted *p*-value). The horizontal dashed line indicates a *p*-value of 0.05. On the right, the red color represents genes upregulated in Δ*cpxR* compared to WT; on the left, the blue color represents genes down-regulated in Δ*cpxR* compared to WT; the gray in the middle represents genes with non-significant differential expression. **(B-H)** qPCR validation of CpxR-upregulated genes. The effect of CpxR on the expression of 7 genes (*KPHS_06860, KPHS_15890, KPHS_16110, KPHS_16120, KPHS_20300, KPHS_28060, KPHS_28080*) identified by RNA-seq was validated through qPCR, respectively. The unpaired, two-sided Student's t-test was performed using the R package (https://www.r-project.org/). Statistical significance was considered when $p \leq 0.05$. * indicates $p < 0.05$; ** indicates $p < 0.01$; *** indicates $p < 0.001$.

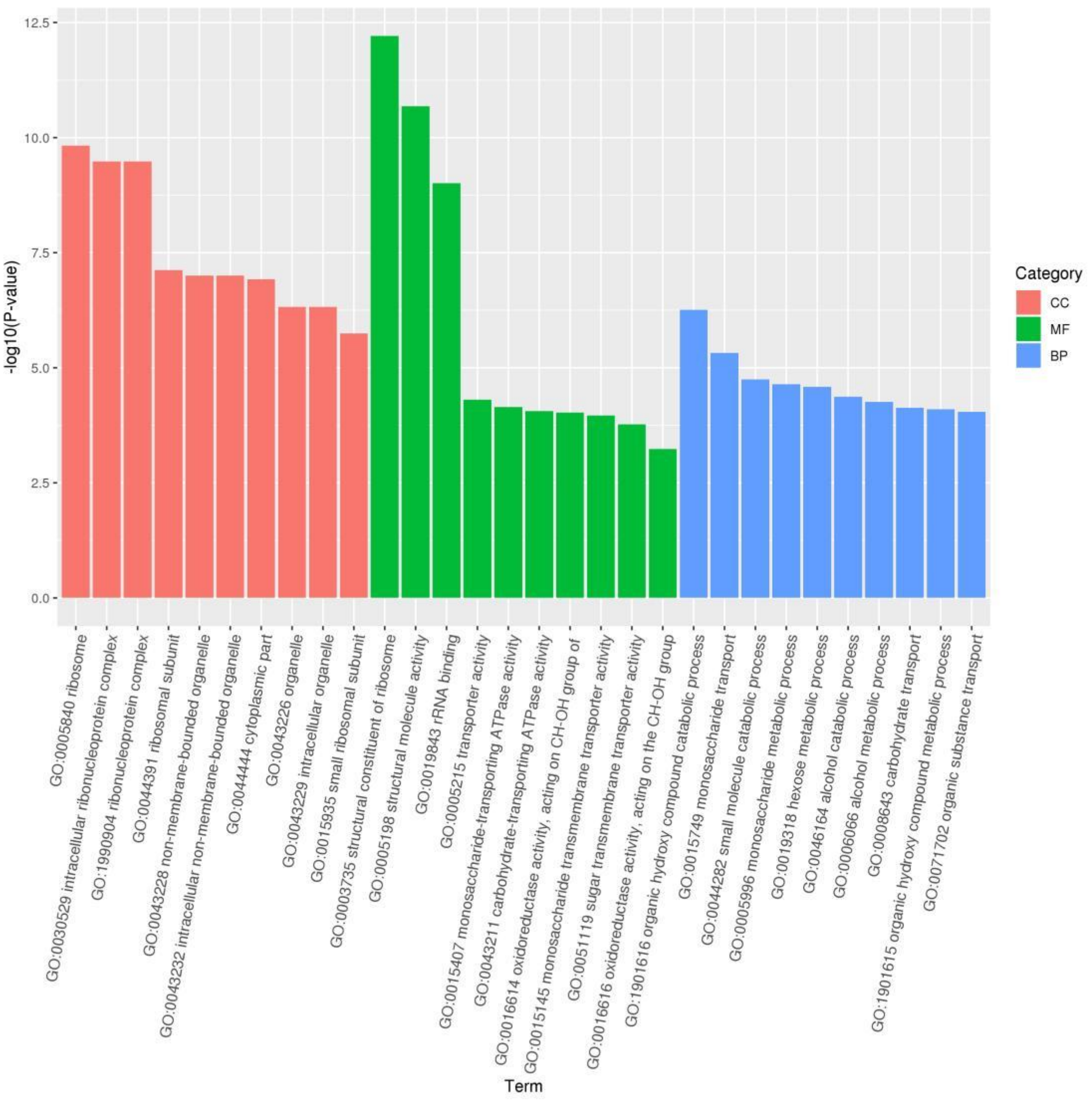

Figure S6. Gene Ontology (GO) enrichment analysis between HS11286 wild-type and Δ*cpxR* mutant. The top enriched GO terms categorized into three groups: Cellular Component (CC) in red, Molecular Function (MF) in green, and Biological Process (BP) in blue. The x-axis represents the GO terms, and the y-axis indicates the significance of enrichment as measured by the -log10(*p*-value).

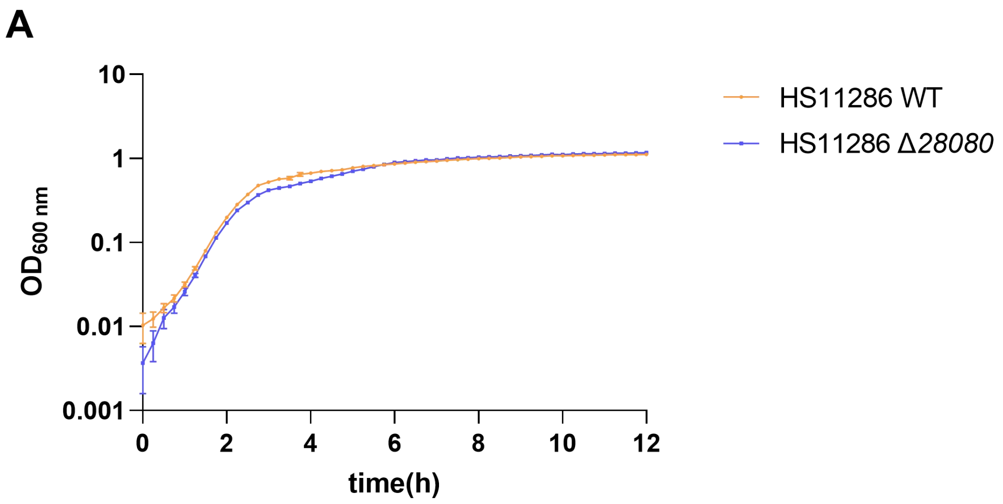

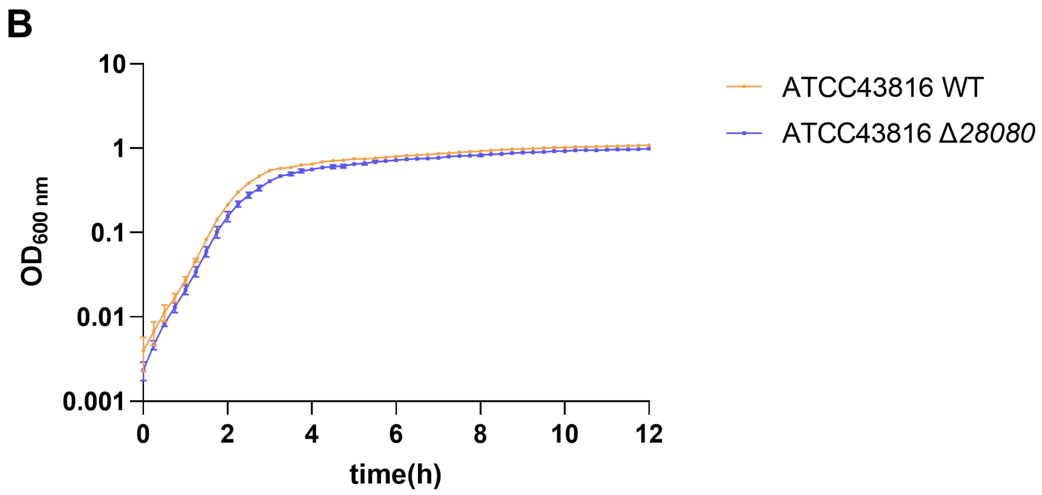

Figure S7. Growth curves of the wild-type and Δ*KPHS_28080* mutants of *K. pneumoniae* HS11286 and ATCC43816.

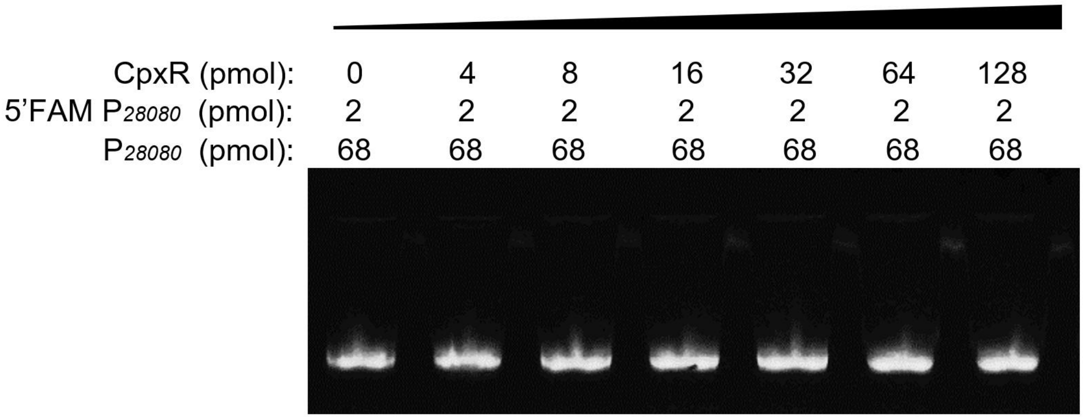

Figure S8. Electrophoretic mobility shift assay of CpxR with 2 pmol FAM-labelled promoter DNA of *KPHS_28080* and 68 pmol unlabelled promoter DNA of *KPHS_28080* (Negative control).

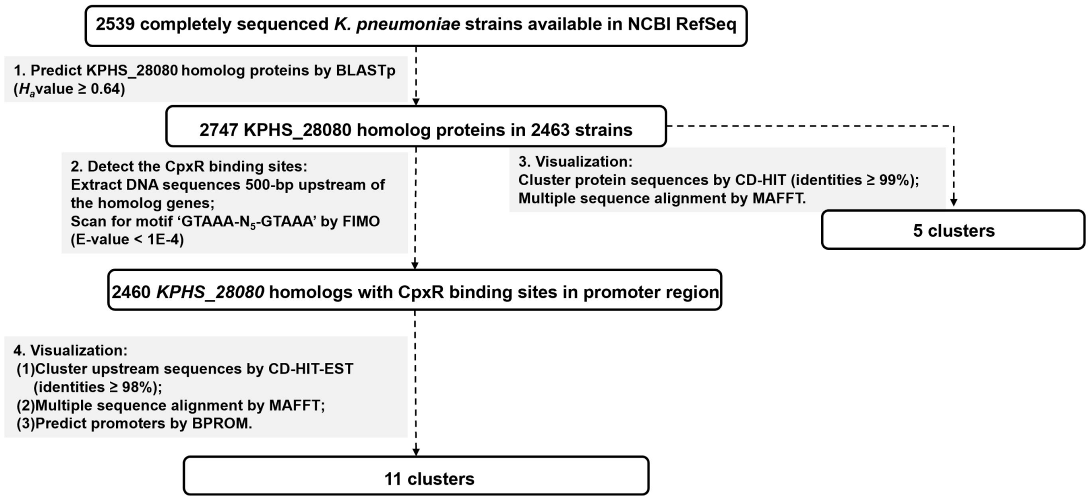

Figure S9. Pipeline for identifying the KPHS_28080 homolog proteins and the CpxR binding sites in the promoter region of *KPHS_28080* homologous genes in the completely sequenced *K. pneumoniae* strains.

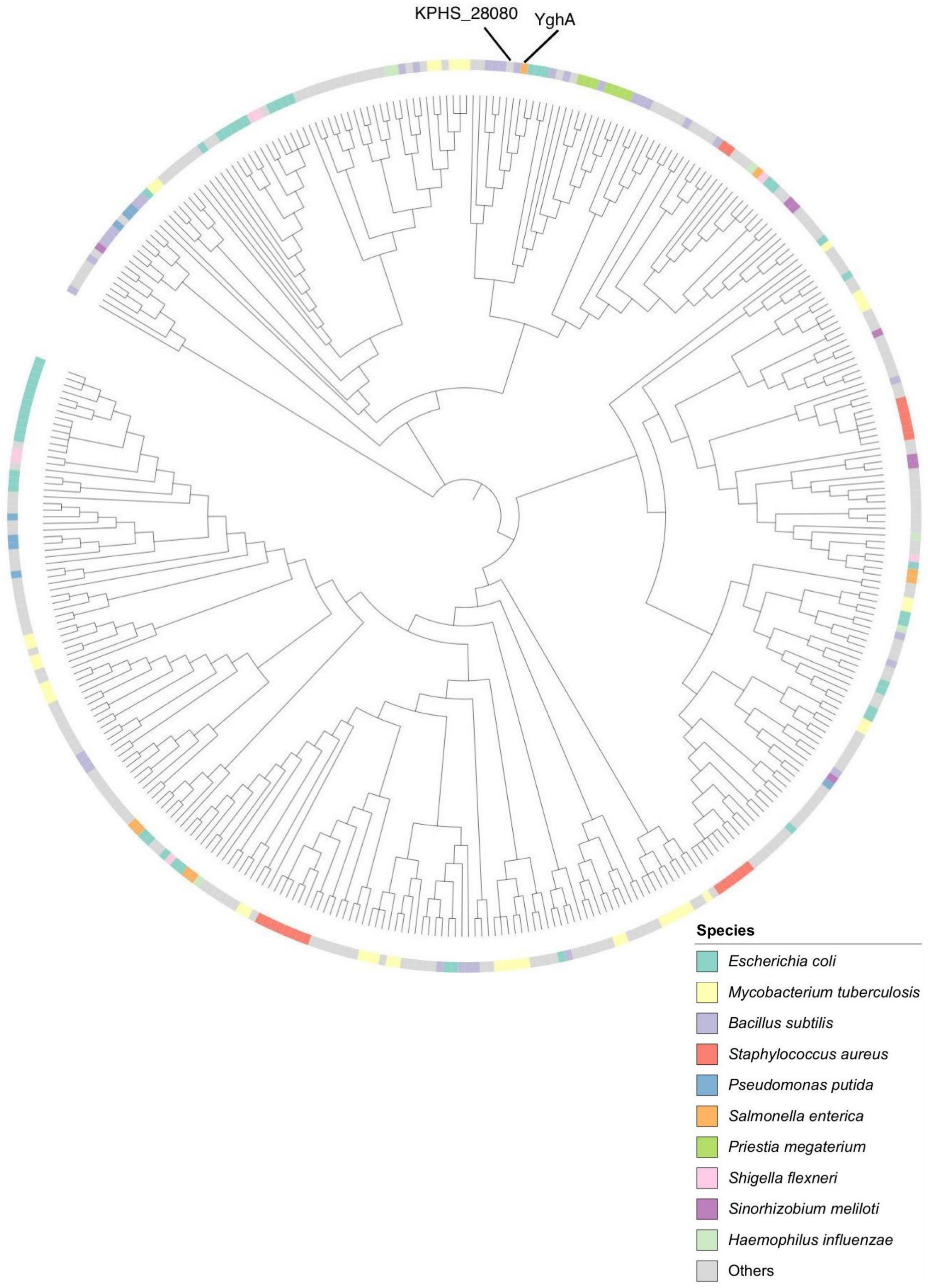

Figure S10. Phylogenetic analysis of the bacterial short-chain dehydrogenase/reductase (SDR) family. The *K. pneumoniae* protein KPHS_28080 (UniProt: A0A0H3GTX5), annotated as a member of the SDR family (InterPro ID: IPR002347), was phylogenetically contextualized. A dataset of 1,153 manually curated SDR family proteins was analyzed, including 382 bacterial homologs. Multiple sequence alignment was performed using MAFFT, followed by evolutionary

tree reconstruction via FastTree under the Maximum Likelihood framework. Reference sequences from *K. pneumoniae* KPHS_28080 and *Salmonella enterica* YghA (UniProt: Q8Z3Q5) are labeled accordingly in the tree.

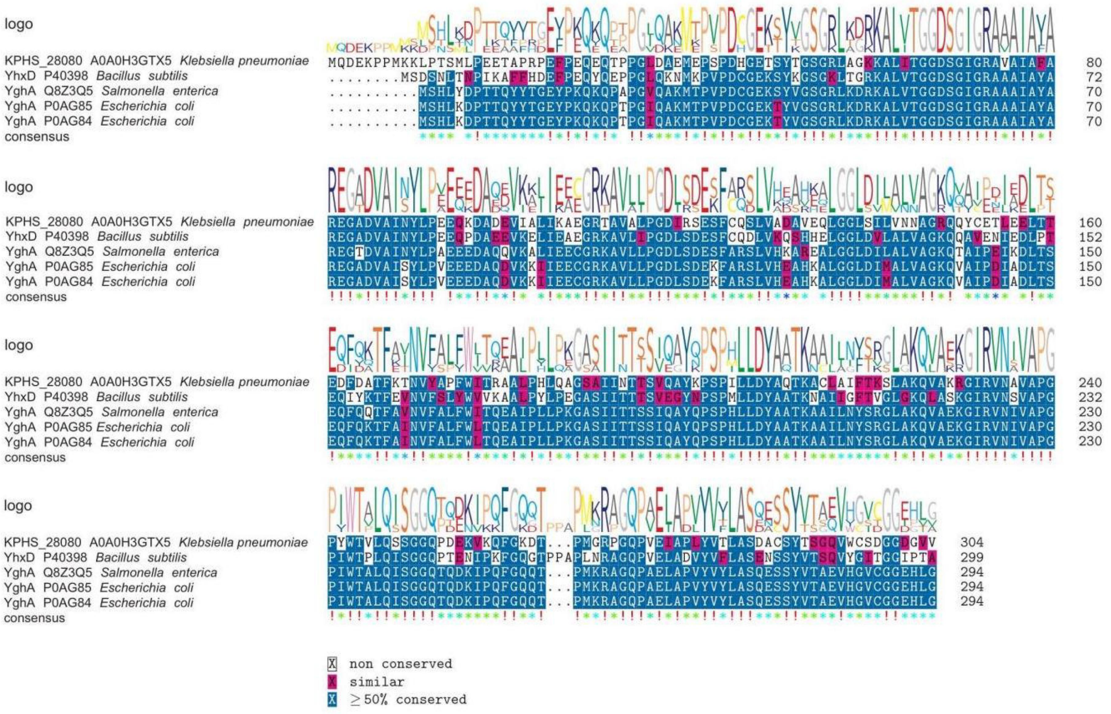

Figure S11. Multiple sequence alignment of proteins within the evolutionary branch containing the KPHS_28080 protein. The evolutionary clade harboring *K. pneumoniae* KPHS_28080 (UniProt: A0AOH3GTX5), identified in Figure S9, comprises five bacterial SDR homologs: *Bacillus subtilis* YhxD (UniProt: P40398), *Salmonella enterica* YghA (UniProt: Q8Z3Q5), and *E. coli* YghA isoforms (UniProt: P0AG84, P0AG85).

Table S1. Strains and plasmids used in this study

Strain	Species	Genotype	Note	Source or Reference
HS11286	*K. pneumoniae*	ST11, K47 serotype, bla_{KPC}^{+}	carbapenem-resistant *K. pneumoniae* clinical strain previously isolated from the sputum specimen in 2011 at Huashan Hospital, Shanghai, China; harbouring six plasmids pKPHS1-pKPHS6, in which the pKPHS2 contains *bla*KPC, Mem^R	(1)
HS11286Δ*cpxR*	*K. pneumoniae*	ST11, K47 serotype, bla_{KPC}^{+}	Derivative of HS11286 with the deletion of *cpxR*, Mem^R	This study
HS11286Δ*cpxR*+*cpxR*	*K. pneumoniae*	ST11, K47 serotype, bla_{KPC}^{+}	Derivative of HS11286 with the deletion of *cpxR,* harbouring plasmid pXG10-Apr-*cpxR*, Mem^R, Apr^R	This study
HS11286Δ*06860*	*K. pneumoniae*	ST11, K47 serotype, bla_{KPC}^{+}	Derivative of HS11286 with the deletion of *KPHS_06860,* Mem^R	This study
HS11286Δ*15890*	*K. pneumoniae*	ST11, K47 serotype, bla_{KPC}^{+}	Derivative of HS11286 with the deletion of *KPHS_15890,* Mem^R	This study
HS11286Δ*16110*	*K. pneumoniae*	ST11, K47 serotype, bla_{KPC}^{+}	Derivative of HS11286 with the deletion of *KPHS_16110,* Mem^R	This study
HS11286Δ*16120*	*K. pneumoniae*	ST11, K47 serotype, bla_{KPC}^{+}	Derivative of HS11286 with the deletion of *KPHS_16120,* Mem^R	This study
HS11286Δ*20300*	*K. pneumoniae*	ST11, K47 serotype, bla_{KPC}^{+}	Derivative of HS11286 with the deletion of *KPHS_20300*, *KPHS_20310*, and *KPHS_20320*. Mem^R	This study
HS11286Δ*28060*	*K. pneumoniae*	ST11, K47 serotype, bla_{KPC}^{+}	Derivative of HS11286 with the deletion of *KPHS_28060* and *KPHS_28070,* Mem^R	This study
HS11286Δ*28080*	*K. pneumoniae*	ST11, K47 serotype, bla_{KPC}^{+}	Derivative of HS11286 with the deletion of *KPHS_28080,* Mem^R	This study
RJ9299	*K. pneumoniae*	ST859, K19 serotype, bla_{KPC}^{+}, $rmpA^{+}$	Hypervirulent carbapenem-resistant *K. pneumoniae*, isolated from Renji Hospital, Shanghai Jiao Tong University	(2)
RJ9299Δ*cpxR*	*K. pneumoniae*	ST859, K19 serotype, bla_{KPC}^{+}, $rmpA^{+}$	Derivative of RJ9299 with the deletion of *cpxR*, Mem^R	This study
RJ9299Δ*cpxR*+*cpxR*	*K. pneumoniae*	ST859, K19 serotype, bla_{KPC}^{+},	Derivative of RJ9299 with the deletion of *cpxR,* harbouring plasmid	This study

		rmpA^{+}	pXG10-Apr-*cpxR*, Mem^{R}, Apr^{R}	
ATCC43816	*K. pneumoniae*	ST493, K2 serotype, *rmpA*$^{+}$	Hypervirulent *K. pneumoniae*, *rmpA*$^{+}$	(3)
ATCC43816Δ*28080*	*K. pneumoniae*	ST493, K2 serotype, *rmpA*$^{+}$	Derivative of ATCC43816 with the deletion of *KPHS_28080, rmpA*$^{+}$	This study
ATCC43816Δ*cpxR*	*K. pneumoniae*	ST493, K2 serotype, *rmpA*$^{+}$	Derivative of ATCC43816 with the deletion of *cpxR, rmpA*$^{+}$	This study
ATCC43816Δ*cpxR*+*cpxR*	*K. pneumoniae*	ST493, K2 serotype, *rmpA*$^{+}$	Derivative of ATCC43816 with the deletion of *cpxR,* harbouring plasmid pXG10-Apr-*cpxR*, Mem^{R}, Apr^{R}	This study
DH5αΔ*cpxR*	*E. coli*		Derivative of DH5α with the deletion of *cpxR* gene	This study

**Plasmid**	**Genotype**	**Note**	**Source or Reference**
pKOBEG-Apr	Expressing lambda Red genes *gam*, *bet* and *exo*	Temperature-sensitive, Apr^{R}	(4)
pFLP2-Apr	Expressing recombinase gene *flp*	Sucrose-sensitive, Apr^{R}	(5)
pXG10-Apr-*cpxR*	pSC101 origin of replication	Low-copy plasmid with *cpxR* used for complementation, Apr^{R}	This study
pXG10-Apr-*KPHS_28080*	pSC101 origin of replication	Low-copy plasmid with *KPHS_28080* used for complementation, Apr^{R}	This study
pET28a(+)-*cpxR*	His-flag	Plasmid for CpxR protein expression, Kan^{R}	This study
pET28a(+)-*cpxR*^{R195H}	His-flag	Plasmid for CpxR R195H protein expression, Kan^{R}	This study
pET28a(+)-*cpxR*^{NTD}	His-flag	Plasmid for CpxR N-terminal domain protein expression, Kan^{R}	This study
pACYC184	p15A origin of replication	Plasmid to construct pAClacZ, Cml^{R}	(6)
pAClacZ	p15A origin of replication	Plasmid for detecting activity of promoter, pACYC184 with RBS before *lacZ* to express β-galactosidase, Cml^{R}	This study
pAClacZ-P_{28080}	p15A origin of replication	Plasmid for detecting activity of *KPHS_28080* promoter, pAClacZ with P_{28080} before *RBS-lacZ*	This study

Abbreviations: Mem^R, meropenem resistance; Apr^R, apramycin resistance; Cml^R, chloramphenicol resistance; RBS, ribosome binding site.

Reference:

1. Liu P, Li P, Jiang X, Bi D, Xie Y, Tai C, *et al*. Complete genome sequence of *Klebsiella pneumoniae* subsp. *pneumoniae* HS11286, a multidrug-resistant strain isolated from human sputum. J Bacteriol. 2012 Apr 1;194(7):1841-1842.

2. Zhu J, Jiang X, Zhao L, Li M. An Outbreak of ST859-K19 Carbapenem-Resistant Hypervirulent *Klebsiella pneumoniae* in a Chinese Teaching Hospital. mSystems. 2022 Jun 28;7(3):e0129721.

3. Appleyard RK. Segregation of New Lysogenic Types during Growth of a Doubly Lysogenic Strain Derived from *Escherichia Coli* K12. Genetics. 1954 Jul 1;39(4):440-452.

4. Chaveroche MK, Ghigo JM, d'Enfert C. A rapid method for efficient gene replacement in the filamentous fungus *Aspergillus nidulans*. Nucleic Acids Res. 2000 Nov 15;28(22):E97.

5. Hoang TT, Karkhoff-Schweizer RR, Kutchma AJ, Schweizer HP. A broad-host-range Flp-FRT recombination system for site-specific excision of chromosomally-located DNA sequences: application for isolation of unmarked *Pseudomonas aeruginosa* mutants. Gene. 1998 May 28;212(1):77-86.

6. Bartolomé B, Jubete Y, Martínez E, de la Cruz F. Construction and properties of a family of pACYC184-derived cloning vectors compatible with pBR322 and its derivatives. Gene. 1991 Jun 15;102(1):75-78.

Table S2. Primers used in this study

Name	Sequence (5'-3')	Description
FRT-hph-F	CCTTAATTAATTGGGGATCTTGAAGTACCTATTCC	Amplifying hygromycin resistance cassette containing FRT sites
FRT-hph-R	AATTAGCTTCAAAAGCGCTCTGAAGTTC	
UHA-cpxR-F	GCAGGTGGTACATCTGGTTGC	Amplifying homologous arms for replacement of *cpxR* in *K. pneumoniae* HS11286, RJ9299, and ATCC43816 with hygromycin resistance cassette containing FRT sites
UHA-cpxR-R	AAGATCCCCAATTAATTAAGGTGTTTAAATACCTCCGAGGCAGAAATTAC	
DHA-cpxR-F	AGAGCGCTTTTGAAGCTAATTTTGGAAGTTTGACTGCACGCAT	
DHA-cpxR-R	GCTGACCAGCATCGTGACG	
cpxR-de-F	GGTAACGACTTCAGCGGCC	Detecting *cpxR* gene in *K. pneumoniae* HS11286, RJ9299, and ATCC43816
cpxR-de-R	GACCAGCATCGTGACGATCAG	
UHA-06860-F	CGGCGCTGATCGACCATAA	Amplifying homologous arms for replacement of *KPHS_06860* in *K. pneumoniae* HS11286 with hygromycin resistance cassette containing FRT sites
UHA-06860-R	AGATCCCCAATTAATTAAGGCATACTTTTCTCCTTACCATAATGGACGG	
DHA-06860-F	GAGCGCTTTTGAAGCTAATTTAGGCGGCCCGCGATGC	
DHA-06860-R	CGCGAACTGCGCTTCGG	
06860-F	GCATTAATTGCTGCGGCTCTG	Detecting *KPHS_06860* gene in *K. pneumoniae* HS11286
06860-R	ACGTCTGCGTCCTGTAAACAG	
UHA-15890-F	CGGCGACTGCAATGACCA	Amplifying homologous arms for replacement of *KPHS_15890* in *K. pneumoniae* HS11286 with

UHA-15890-R	AGATCCCCAATTAATTAAGGTAACACATGAGGTGGGCAGATCG	hygromycin resistance cassette containing FRT sites
DHA-15890-F	GAGCGCTTTTGAAGCTAATTCATCGGATACTCCTGTTAAGCCG	
DHA-15890-R	GCTGCCGGATCGTGAATAG	
15890-F	GTTGTCTTGGGTCGTATTGCCG	Detecting *KPHS_15890* gene in *K. pneumoniae* HS11286
15890-R	CGCCGAACAACGTGGACAA	
UHA-16110-F	GCAGCGTCGTTACGGTCTAC	Amplifying homologous arms for replacement of *KPHS_16110* in *K. pneumoniae* HS11286 with hygromycin resistance cassette containing FRT sites
UHA-16110-R	AGATCCCCAATTAATTAAGGTAACCCAGCACTCAAGCCCT	
DHA-16110-F	GAGCGCTTTTGAAGCTAATTCATTTTTTTGCCTCAGTTAGCCGA	
DHA-16110-R	CAATCTCATGCATTTCGCCAGC	
16110-F	GGTTTAGCGACCGGCCAAT	Detecting *KPHS_16110* gene in *K. pneumoniae* HS11286
16110-R	AGATATTTCTGCATCAACCCACCC	
UHA-16120-F	CAGCATCCATTTTCACGGCC	Amplifying homologous arms for replacement of *KPHS_16120* in *K. pneumoniae* HS11286 with hygromycin resistance cassette containing FRT sites
UHA-16120-R	AGATCCCCAATTAATTAAGGTAAATAAACCCACTGTGCTGGCAG	
DHA-16120-F	GAGCGCTTTTGAAGCTAATTCATAGCTCGAACTCTCCCACGT	
DHA-16120-R	GTCCTCAAGGCCTGTCCAGG	
16120-F	GTACAGCATATCCAGCGCCT	Detecting *KPHS_16120* gene in *K. pneumoniae* HS11286
16120-R	CCATTCCAGCAGAGTTTGTCTTC	
UHA-20300-F	GCCGCAGAGGTGATCTGC	Amplifying homologous arms for replacement of *KPHS_20300, KPHS_20310* and *KPHS_20320* in *K.*

UHA-20300-R	AGATCCCCAATTAATTAAGGCATAGAAATACCTTCTGGAGTCAGAAAATAACTG	*pneumoniae* HS11286 with hygromycin resistance cassette containing FRT sites
DHA-20300-F	GAGCGCTTTTGAAGCTAATTTGATGTTCGCATGAGCCCCG	
DHA-20300-R	CCTGCGGCGTCTGCATC	
20300-F	CTCAGCGCGGCGTTCAA	Detecting *KPHS_20300* gene in *K. pneumoniae* HS11286
20300-R	CCGTGCTCTCAACGACTTCA	
UHA-28060-F	CGCCAGGCATATCGGTATCC	Amplifying homologous arms for replacement of *KPHS_28060* and *KPHS_28070* in *K. pneumoniae* HS11286 with hygromycin resistance cassette containing FRT sites
UHA-28060-R	AGATCCCCAATTAATTAAGGTAAAAGGTTAATGATCAGAGGTGTATATGACGG	
DHA-28060-F	GAGCGCTTTTGAAGCTAATTCATAATTTTCACCTTTGGTTTGATCAATATCGG	
DHA-28060-R	GGATAGCGTTGCGTTATTATTGCTG	
28060-F	GCTCGCGCTGCGCTATAT	Detecting *KPHS_28060* gene in *K. pneumoniae* HS11286
28060-R	AGCGCTGTCATCTTGTCGC	
UHA-28080-F	CAGATACCCATCCGCGCAC	Amplifying homologous arms for replacement of *KPHS_28080* in *K. pneumoniae* HS11286 and ATCC43816 with hygromycin resistance cassette containing FRT sites
UHA-28080-R	AGATCCCCAATTAATTAAGGCATAGTCATCTCCTTCAGACAGAACGG	
DHA-28080-F	GAGCGCTTTTGAAGCTAATTTAAGCCTCCCGGCATACCGA	
DHA-28080-R	CTTGATGCGCTGCAGAGG	
28080-F	GACTCCGGTATTGGCCGTGC	Detecting *KPHS_28080* in *K. pneumoniae* HS11286 and ATCC43816
28080-R	GGATTCGGAGCGAATATCACCC	
pKOBEG-F	CCCGCTAGCGAAAAGATGTTTCGTGAAGC	Detecting λ-Red related gene on pKOBEG-Apr

pKOBEG-R	GGGAAGCTTATTATCGTGAGGATGCGTCA	
pFLP2-F	TGCTCTAGAGCACGGCATTTTCTTTTGCGTTT	Detecting *sacB* gene on pFLP2-Apr
pFLP2-R	CGCGGATCCGCGTCTTTAGGCCCGTAGTCTGC	
pXG10-Apr-FU-F	TCTAGAGGCATCAAATAAAACGAAAGGC	Amplifying pXG10 plasmid to construct plasmid pXG10-Apr-*cpxR*, pXG10-Apr-$cpxR^{R195H}$, and pXG10-Apr-$cpxR^{NTD}$
pXG10-Apr-FU-R	GACGTCGATATCTGGCGAAAATGAG	
cpxR-101-FU-F	CCAGATATCGACGTCCACATACTCCCAAAACGTTTGTGTCG	Amplifying *cpxR* to construct plasmid pXG10-Apr-*cpxR*
cpxR-101-FU-R	TTTGATGCCTCTAGATCATGAAGCGGAAACCATCAGA	
28080-pXG10-FU-F	TTTCGCCAGATATCGACGTCGCTCTGCCATAATTTTCACCTTTGG	Amplifying *KPHS_28080* to construct plasmid pXG10-Apr-*KPHS_28080*
28080-pXG10-FU-R	TTTTATTTGATGCCTCTAGATTAGACCACACCATCCCCGC	
cpxR-pET28-FU-F	CCGCGCGGCAGCCATATGAATAAAATCCTGTTAGTTGATGATGACCGG	Amplifying *cpxR*, $cpxR^{R195H}$to construct plasmid pET28a(+)-*cpxR*, pET28a(+)-$cpxR^{R195H}$
cpxR-pET28-FU-R	CGGCCGCAAGCTTTCATGAAGCGGAAACCATCAGA	
cpxR-Re-FU-F	CCGCGCGGCAGCCATATGAATAAAATCCTGTTAGTTGATGATGACCGG	Amplifying $cpxR^{NTD}$ to construct plasmid pET28a(+)-$cpxR^{NTD}$
cpxR-Re-FU-R	TGCGGCCGCAAGCTTTCAGCCGGCTTCGGTCG	
cpxR-R195H-F	CGACCACGCCATCGATATGCATATTTCCAACC	Constructing mutation R195H
cpxR-R195H-R	TCGATGGCGTGGTCGAACGGCGTCAGG	

RBSlacZ-F	CTCGAGGGATCCAGGAGGAATTCACCATGACCATGATTACGGATTCACTG	Amplifying RBS and *lacZ* to construct plasmid pAClacZ
lacZter-R	AATGGATTTCCTTACGCGAAATACGG	
pACYC184-F	GTAAGGAAATCCATTTTTATCAGGCTCTGGGAGGCAG	Amplifying pACYC184 to construct plasmid pAClacZ
pACYC184-R	CCTGGATCCCTCGAGGCAATTTAACTGTGATAAACTACCGC	
KpgapA-qR	CCTGACCGACGAAACCG	Detect the expression of *gapA* gene
KpgapA-qF	GCGTTGGAAACGATGTCCTG	
06860-qF	TCATTGCATTAATTGCTGCGG	Detect the expression of *KPHS_06860* gene
06860-qR	GTAAACAGGCTCACCAGGAAC	
15890-qF	CTTAGGTCTTGCCAGCGGC	Detect the expression of *KPHS_15890* gene
15890-qR	CTATTGTCCACGTTGTTCGGC	
16110-qF	CACCAGCGACAATTATTTCGCG	Detect the expression of *KPHS_16110* gene
16110-qR	CTCTTCCGGCGTCGAGTTGG	
16120-qF	CCATTCCAGCAGAGTTTGTCTTC	Detect the expression of *KPHS_16120* gene
16120-qR	GCAGACGATGCTGGATTTCCG	
20300-qF	CCAGATCGAACGTATCGACCAG	Detect the expression of *KPHS_20300* gene
20300-qR	CGGCAATTAATGCAGCATCACG	
28060-qF	GCGCTGTCATCTTGTCGCC	Detect the expression of *KPHS_28060* gene

28060-qR	GCGCTATATCCGGATAAATGGCC	
28080-qF	GACTCCGGTATTGGCCGTGC	Detect the expression of *KPHS_28080* gene
28080-qR	GGATTCGGAGCGAATATCACCC	

Abbreviations: FRT, flippase recognition sites

Table S3. Seven selected genes with significantly down-regulated expression after CpxR deletion in *K. pneumoniae* HS11286.

Gene	Product	Log_2FoldChange(Δ*cpxR*/WT)
KPHS_06860	DUF1328 domain-containing protein	-7.66
KPHS_15890	TPA: hypothetical protein &YbgS-like family protein	-7.46
KPHS_16110	LuxR transcriptional regulator	-7.5
KPHS_16120	Biofilm development regulator YmgB/AriR family protein	-7.91
KPHS_20300	Putative structural protein	-9.74
KPHS_28060	Hypothetical protein	-8.16
KPHS_28080	**SDR family oxidoreductase**	**-9.05**
